# *CaSBP11* Participates in the Defense Response of Pepper to *Phytophthora capsici* through Regulating the Expression of Defense-Related Genes

**DOI:** 10.3390/ijms21239065

**Published:** 2020-11-28

**Authors:** Huai-Xia Zhang, Xiao-Hui Feng, Jing-Hao Jin, Abid Khan, Wei-Li Guo, Xiao-Hua Du, Zhen-Hui Gong

**Affiliations:** 1 College of Horticulture, Northwest A&F University, Yangling, Shaanxi 712100, China; 2016060124@nwsuaf.edu.cn (H.-X.Z.); fengxiaohui@ibcas.ac.cn (X.-H.F.); jinghao.jin@yzu.edu.cn (J.-H.J.); 2 School of Horticulture Landscape Architecture, Henan Institute of Science and Technology, Xinxiang 453003, China; guoweili1226@hist.edu.cn (W.-L.G.); duxhb313@gmail.com (X.-H.D.); 3 Department of Horticulture, The University of Haripur, Haripur 22620, Pakistan; abidagriculturist@gmail.com

**Keywords:** pepper, *CaSBP11*, *Phytophthora capsici*, defense-related genes, *Nicotiana benthamiana*

## Abstract

Squamosa promoter binding protein (SBP)-box genes are plant-specific transcription factors involved in plant growth and development, morphogenesis and biotic and abiotic stress responses. However, these genes have been understudied in pepper, especially with respect to defense responses to *Phytophthora capsici* infection. *CaSBP11* is a SBP-box family gene in pepper that was identified in our previous research. Silencing *CaSBP11* enhanced the defense response of pepper plants to *Phytophthora capsici*. Without treatment, the expression of defense-related genes (*CaBPR1*, *CaPO1*, *CaSAR8.2* and *CaDEF1*) increased in *CaSBP11*-silenced plants. However, the expression levels of these genes were inhibited under transient *CaSBP11* expression. *CaSBP11* overexpression in transgenic *Nicotiana benthamiana* decreased defense responses, while in *Arabidopsis*, it induced or inhibited the expression of genes in the salicylic acid and jasmonic acid signaling pathways. *CaSBP11* overexpression in *sid2-2* mutants induced *AtNPR1*, *AtNPR3*, *AtNPR4*, *AtPAD4*, *AtEDS1*, *AtEDS5*, *AtMPK4* and *AtNDR1* expression, while *AtSARD1* and *AtTGA6* expression was inhibited. *CaSBP11* overexpression in *coi1-21* and *coi1-22* mutants, respectively, inhibited *AtPDF1.2* expression and induced *AtPR1* expression. These results indicate *CaSBP11* has a negative regulatory effect on defense responses to *Phytophthora capsici*. Moreover, it may participate in the defense response of pepper to *Phytophthora capsici* by regulating defense-related genes and the salicylic and jasmonic acid-mediated disease resistance signaling pathways.

## 1. Introduction

Pepper (*Capsicum annuum* L.) is an important crop that used as a vegetable, spice, food colorant and source of medicinal compounds [1]. It is rich in vitamins and minerals and has a high nutritional and economic value [2]. However, it is easily infected by *Phytophthora capsici* (*P. capsici*) during its cultivation, causing the occurrence of *Phytophthora blight*, which seriously affects its production and economic benefits. *P. capsici* is a soil-borne pathogen that can infect various tissues of pepper plants (roots, stems, leaves, flowers and fruits) as well as other crop plants, such as tomato, eggplant, cucurbits (including cucumber, honeydew, watermelon and pumpkin), snap peas and lima beans [3,4]. To combat pathogen infection, plants have evolved a series of defense mechanisms, including the regulation by transcription factors and hormones that enhance resistance to different stresses [5]. Transcription factors play an important role in the process of transforming external signals into intracellular signals, thus inducing specific hormone signaling pathways and gene expression cascades that activate defense-related targets [6]. For example, *AtWRKY33* plays a positive role in *Arabidopsis* defense responses against *Botrytis cinerea* infection through reprogramming the expression of camalexin biosynthesis genes [7]. *AtWRKY18*, a pathogen- and salicylic acid-induced *Arabidopsis* transcription factor, enhanced resistance to infections by the bacterial pathogen *Pseudomonas syringae* by positively regulating the expression of defense-related genes [6,8]. *CaAP2/ERF064* plays a positive role in plant defense responses against *P. capsici* infection by modulating the transcription of pathogenesis-related (PR) genes in pepper [9].

Squamosa promoter binding protein (SBP)-box genes are plant specific transcription factors with a conserved SBP domain, which consists of about 76 amino acid residues and contains two typical zinc finger structural proteins (C3H and C2HC). In addition, there is a highly conserved nuclear localization signal (NLS) at the C-terminus of the conserved SBP domain, which partially overlaps with the C2HC zinc finger sequence [10,11]. The SBP-box genes were first discovered in *Antirrhinum majus* and named *AmSBP1* and *AmSBP2* according to their ability to bind to the promoter of the *Antirrhinum majus* floral meristem identity gene SQUAMOSA [10]. Subsequently, SBP has been isolated from *Arabidopsis thaliana* [12], *Betula platyphylla* [13], tomato [14], apple [15], *Salvia miltiorrhiza* [16], *Gossypium hirsutum* [17], pepper [18], Chinese Jujube [19], *Petunia* [20], *Euphorbiaceae* [21] and other plants [22,23,24,25,26,27].

As the number of isolated SBP-box genes has increased, researchers have studied the function of more SBP-box genes from different species. For example, knockout of *SPL8* can affect the occurrence of megaspores, the formation of trichomes and the elongation of stamen filaments in *Arabidopsis thaliana* [28]. In rice, overexpression of *TaSPL20* and *TaSPL21* can promote panicle branching and *TaSPL21*-overexpression lines can exhibit increased thousand-grain weights [29]. In *Medicago sativa, SPL13* negatively regulates its target gene *MYB112* and regulates the branching and flowering time [30].

As research on the function of SBP genes continues, more and more studies have shown that SBP genes play important roles in biotic stress responses. *AtSPL14* is involved in plant growth and development in *Arabidopsis thaliana* and is sensitive to fumonisin B1, which can induce programmed cell death [31]. *NbSPL6* is required in the process of tobacco mosaic resistance mediated by N in tobacco. In addition, *AtSPL6*, a homolog of *NbSPL6* in *Arabidopsis thaliana*, is essential for resistance to *Pseudomonas syringae* infection, as mediated by toll and interleukin-1 receptor nucleotide binding-leucine rich repeat (TIR-NB-LRR) [32]. In *Arabidopsis,* AtSPL9 interacts with JAZ protein and negatively regulates the jasmonic acid signaling pathway and resistance to insect infection by promoting the accumulation of JAZ3 [33]. Similarly, AtJMT negatively regulates the expression of *AtSPL2* and participates in the jasmonic acid-mediated disease resistance signaling pathway [34]. In rice, *OsSPL9* can bind to the specific motif (cure element) in the promoter region of miR528 thus activating its transcription and accumulation, inhibit the expression of the target mRNA Ao and ultimately relieve the inhibition of ascorbic acid oxidase (AO) on rice stripe virus [35]. In grape, *VpSBP5* may regulate resistance to powdery mildew through the jasmonic acid- and salicylic acid-mediated disease resistance signaling pathways [36]. However, the role of SBP-box genes in pepper has been relatively ignored, especially in the context of responses to *P.capsici* infection.

*CaSBP11* (accession no. Capana10g000709), which has a 1524bp-nucleotide open reading frame encoding 507 amino acids, is a SBP-box gene in pepper that encodes all the features of typical SBP-box proteins [18]. Moreover, it responds to infection by compatible and incompatible strains of *P. capsici* [18]. In addition, the expression of *CaSBP11* can be induced by salicylic acid (SA) and methyl jasmonate (MeJA) and is inhibited in the early stage by SA and MeJA synthesis inhibitors [18]. Therefore, we selected this gene to further elucidate its function and mechanism of action in plant defense responses against *P. capsici* infection in this study.

## 2. Results

### 2.1. CaSBP11 Protein Localizes to the Nucleus

**Figure 1:**
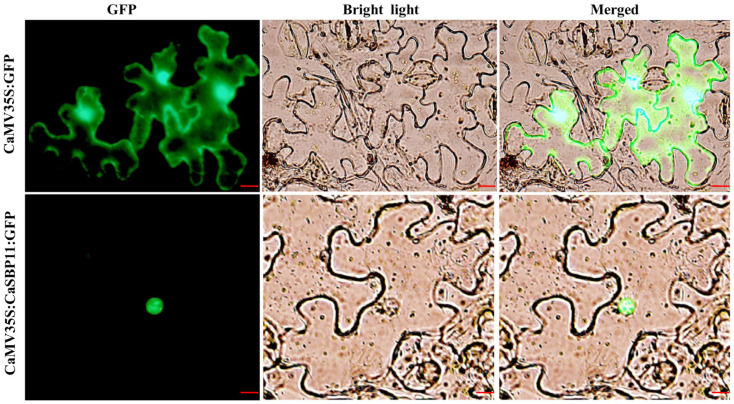
Subcellular localization of the CaSBP11 protein. *Agrobacterium tumefaciens* strain GV3101 was used to transiently express the CaMV35S:CaSBP11:GFP and CaMV35S:GFP (used as a control) in *N. benthamiana* leaves. The fluorescence was visualized using a laser scanning confocal microscope under bright and fluorescent fields. The photographs were taken in a dark field for green fluorescence and under bright light for the morphology of the cell. Merged is a superimposed picture of bright field and dark field. Bars in this picture are 50 μm.

To assess the subcellular localization of CaSBP11 protein in pepper, *Agrobacterium tumefaciens* GV3101 carrying CaMV35S:CaSBP11:GFP or CaMV35S:GFP (used as a control) constructs were transiently expressed in the leaves of *Nicotiana benthamiana (N. benthamiana)*. In transformed leaves, the nucleus, cytoplasm and cell membrane of control plants showed green fluorescence signals. However, the green fluorescence signal only appeared in the cell nuclei of plants expressing CaMV35S:CaSBP11:GFP (Figure 1), indicating that CaSBP11 protein is localized in the nucleus.

### 2.2. Silencing of CaSBP11 Enhanced Pepper Resistance to P. capsici Infection

**Figure 2:**
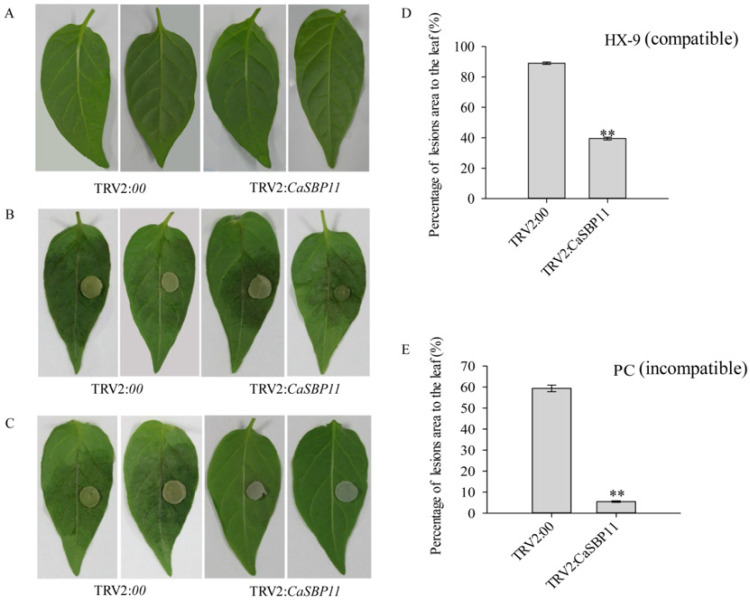
Disease resistance identification of the detached leaves of CaSBP11 silencing in pepper. (**A**) Phenotypes of detached leaves of CaSBP11-silenced and negative control plants without inoculation with *P. capsici* three days. (**B**) Phenotypes of detached leaves of CaSBP11-silenced and negative control plants after inoculation with compatible *P. capsici* three days. (**C**) Phenotypes of detached leaves of CaSBP11-silenced and negative control plants after inoculation with incompatible *P. capsici* three days. (**D**) Percentage of lesions area to the leaf of CaSBP11-silenced and negative control plants after inoculation with compatible *P. capsici* three days. Data were collected from twelve leaves of CaSBP11-silenced and negative control plants, respectively. (**E**) Percentage of lesions area to the leaf of CaSBP11-silenced and negative control plants after inoculation with incompatible *P. capsici* three days. Data were collected from fourteen leaves of CaSBP11-silenced and negative control plants, respectively. The diameter of the plug in (**B**) and (**C**) is 4mm. The means were analyzed using the least significant difference (LSD). ** represents a significant difference at *p <* 0.01. Mean values and SDs are shown.

To further study the role of *CaSBP11* in the defense response of pepper plants to *P. capsici* infection, *CaSBP11* was silenced. In this study, *CaPDS* (phytoene desaturase, GenBank accession number, X68058), which induces a leaf photo-bleaching phenotype when silenced, was selected as a positive control. Additionally, the empty TRV2:*00* vector was selected as a negative control. Forty days after injection, the positive control plants showed photo-bleaching, while the TRV2:*00* and the CaSBP11-silenced plants exhibited no obvious phenotypic changes (Supplementary Materials, Figure S1A). Subsequently, the silencing efficiency of *CaSBP11* was checked by qPCR. As shown in Supplementary Materials, Figure S1B, the silencing efficiency of *CaSBP11* was 82.3%. Then, the disease resistance of CaSBP11-silenced plants was assessed. After 3 days in culture conditions without *P. capsici* inoculation, there was no obvious phenotypic difference between the detached leaves of CaSBP11-silenced plants and negative control plants (Figure 2A). However, 3 days after inoculation with compatible *P. capsici* (HX-9), the detached leaves of *CaSBP11*-silenced plants appeared to develop hygrophanous lesions, with lesion areas significantly smaller than that of the negative control leaves (Figure 2B,D). Moreover, 3 days after inoculation with incompatible *P. capsici* (PC), the area of diseased leaves of CaSBP11-silenced plants was significantly smaller than that of the negative control plants (Figure 2C,E). In addition, 3 days after inoculation with compatible *P. capsici* (HX-9), the lesion area of CaSBP11-silenced plants and negative control plants were larger than those inoculated with incompatible *P. capsici* (PC) (Figure 2).

**Figure 3:**
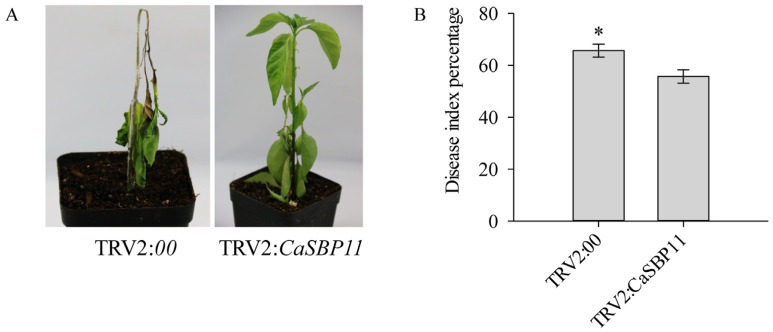
Phenotypes and disease index percentage of the CaSBP11-silenced and negative control plants after inoculation with compatible *P. capsici* (HX-9) sixteen days. (**A**) Phenotypes of the silenced and negative control plants after inoculation with the strain of *P. capsici* (HX-9) sixteen days. The diameter of the pot in (A) is 7cm. (**B**) Disease index percentage of the CaSBP-silenced and negative control plants after being inoculated with the strain of *P. capsici* (HX-9) sixteen days. The means were analyzed using the least significant difference (LSD). * represents significant differences at *p <* 0.05. Mean values and SDs for at least three replicates are shown.

Sixteen days after inoculation with *P. capsici* (HX-9, the lower leaves of the CaSBP11-silenced plants wilted and the stem base turned black (Figure 3A). However, the leaves in the lower layer of the negative control plants dropped off, the stem base became black and the entire plant wilted (Figure 3A). The disease phenotypes of CaSBP11-silenced and control plants were categorized into five different levels (level 0, no symptoms; level 1, the lower leaves of the plant turn yellow or wilt; level 2, the lower leaves of the plant exhibit an obvious deciduous phenomenon or the whole plant is wilted; level 3, blackening of stem base and all leaves have fallen off except at growth points; level 4, the whole plant is dead. Supplementary Materials, Figure S2). Then, the disease phenotypes of CaSBP11-silenced and control plants were quantified. As shown in Figure 3B, the disease index percentage of negative control plants was significantly higher than that of the CaSBP11-silenced plants. These results indicate that *CaSBP11* silencing enhanced pepper resistance to *P. capsici* infection.

### 2.3. CaSBP11 Is Involved in Pepper Defense Response to P. capsici through Inducing Defense-Related Genes

**Figure 4:**
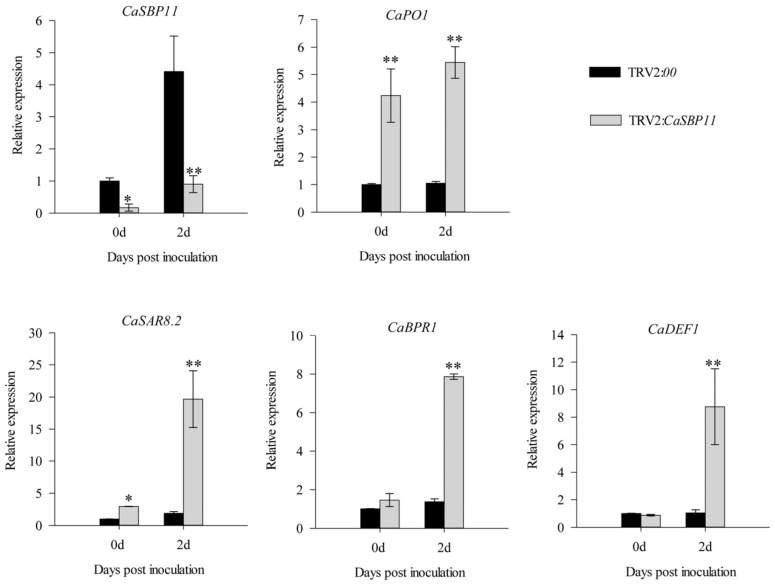
The expression of defense-related genes after inoculation with compatible *P. capsici* (HX-9) in *CaSBP11*-silenced and negative control plants. In this experiment, roots from *CaSBP11*-silenced and negative control plants were collected at 0 days (collected at the time of inoculation) and 2 days, for the detection of defense-related genes. The means were analyzed using the least significant difference (LSD). * and ** represent significant differences at *p <* 0.05 and *p <* 0.01 respectively. Mean values and SDs for three replicates are shown.

**Figure 5:**
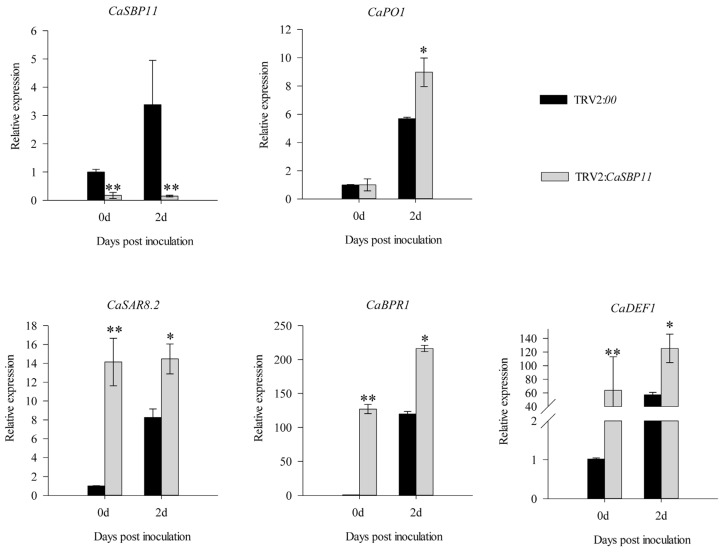
The expression of defense-related genes after inoculation with incompatible *P. capsici* (PC) in CaSBP11-silenced and negative control plants. In this experiment, roots from *CaSBP11*-silenced and negative control plants were collected at 0 days (collected at the time of inoculation) and 2 days, for the detection of defense-related genes. The means were analyzed using the least significant difference (LSD). * and ** represent significant differences at *p <* 0.05 and *p <* 0.01 respectively. Mean values and SDs for three replicates are shown.

*P. capsici* is a soil borne disease. Therefore, in this study, we inoculated compatible (HX-9) and incompatible (PC) *P. capsici* into CaSBP11-silenced and negative control plants by root irrigation and then detected the expression levels of defense-related genes (Pepper peroxidase-like gene, *CaPO1*, accession number AF053343; Pepper systemic acquired resistance gene, *CaSAR8.2*, accession number AF112868; Pepper pathogenesis-related (PR)-1 protein, *CaBPR1,* accession number AF053343; Pepper defensin gene, *CaDEF1*, accession number AF442388). After inoculation with compatible (HX-9) *P. capsici*, the expression of *CaSBP11* in CaSBP11-silenced and negative control plants increased at 2 days compared with that at 0 days. However, the expression level in the CaSBP11-silenced plants was significantly lower than that in the negative control plants (Figure 4). In addition, the expression levels of defense-related genes (*CaPO1*, *CaSAR8.2*, *CaBPR1 and CaDEF1*) in CaSBP11-silenced plants were significantly higher than those in negative control plants at 2 days (Figure 4). Notably, the expression levels of *CaPO1* and *CaSAR8.2* in the CaSBP11-silenced plants were significantly higher than those in the negative control plants at 0 days (Figure 4). Two days after inoculation with incompatible (PC) *P. capsici*, the expression level of *CaSBP11* in the CaSBP11-silenced plants was significantly lower than that in the negative control plants (Figure 5). In addition, the expression levels of the defense-related genes *CaPO1*, *CaSAR8.2*, *CaBPR1 and CaDEF1* in CaSBP11-silenced plants and negative control plants were increased at 2 days compared with those at 0 days, while the expression levels in the CaSBP11-silenced plants were significantly higher than those in the negative control plants at 2 days (Figure 5). Moreover, the expression levels of *CaSAR8.2*, *CaBPR1 and CaDEF1* in the CaSBP11-silenced plants were significantly higher than those in the negative control plants at 0 days (Figure 5). Notably, the expression level of defense related genes (except *CaSAR8.2*) after inoculation with incompatible *P. capsici* (PC) was much higher than that after inoculation with compatible *P. capsici* (HX-9). These results indicated that *CaSBP11* is involved in pepper defense responses against *P. capsici* through the regulation of defense-related genes.

### 2.4. Transient Expression of CaSBP11 in Pepper

**Figure 6:**
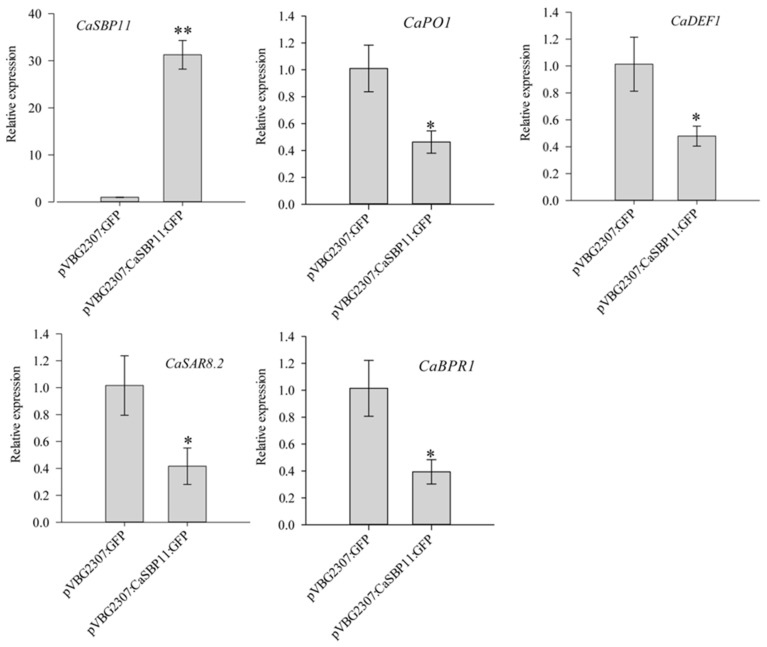
Transient expression of *CaSBP11* in pepper. Leaves were collected at 2 days after *Agrobacterium* (carrying the pVBG2307:CaSBP11:GFP and pVBG2307:GFP) infection and used for the detection of gene expression. pVBG2307:CaSBP11:GFP represents the *CaSBP11* transient expression plants and pVBG2307:GFP represents control plants. The means were analyzed using the least significant difference (LSD). * and ** represent significant differences at *p <* 0.05 and *p <* 0.01 respectively. Mean values and SDs for three replicates are shown.

In the above results, it was found that the expression of the defense-related genes *CaPO1*, *CaSAR8.2*, *CaBPR1 and CaDEF1* was increased in CaSBP11-silenced plants. Thus, we speculated that *CaSBP11* may inhibit the expression of these defense-related genes. To test this hypothesis, *CaSBP11* was transiently expressed in pepper. In this experiment, leaves were collected after *Agrobacterium* (carrying the pVBG2307:CaSBP11:GFP and pVBG2307:GFP constructs) infection two days for the detection of gene expression. As shown in Figure 6, *CaSBP11* was successfully transiently expressed in pepper plants and its expression level in transient expression plants (transformed with hpVBG2307:CaSBP11:GFP constructs) was significantly higher than that in control plants (pVBG2307:GFP). In addition, the expression levels of the defense-related genes *CaPO1*, *CaSAR8.2*, *CaBPR1 and CaDEF1* in plants transiently expressing *CaSBP11* were significantly lower than those in control plants. These results indicate that *CaSBP11* negatively regulates the expression of defense-related genes. Thus, we speculated that *CaSBP11* is a negative regulator of plant immunity.

### 2.5. Overexpression of CaSBP11 in N. benthamiana Increased Susceptibility to P. capsici Infection

**Figure 7:**
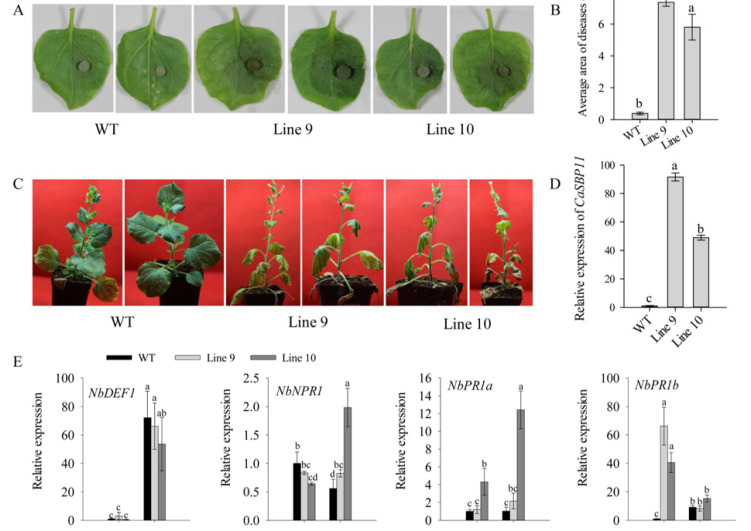
Disease resistance identification of *CaSBP11* transgenic lines (Lines 9 and 10) in *N. benthamiana*. (**A**) Phenotypes of the detached leaves of transgenic and WT plants after inoculation with compatible *P. capsici* two days. The diameter of the plug in (**A**) is 4mm. (**B**) The average diseased areas of the detached leaves of transgenic and WT plants after inoculation with compatible *P. capsici* two days. (**C**) Phenotypes of transgenic and WT plants after inoculation with compatible *P. capsici* six days. The diameter of the plot in (**C**) is 7 cm. (**D**) The expression level of *CaSBP11* in transgenic and WT plants. (**E**) The expression of defense-related genes in transgenic and WT plants after inoculation with compatible *P. capsici*. Leaves of the *CaSBP11* transgenic and WT plants were collected at 0 days (collected at the time of inoculation) and 3 days, for the detection of defense-related genes. The means were analyzed using the least significant difference (LSD). Different small letters (a, ab, b, bc, c, cd, d) represent significant differences as determined by the LSD test (*p* < 0.05). Mean values and SDs for at least three replicates are shown.

To further study the function of *CaSBP11* in the defense response to *P. capsici* infection, it was overexpressed in *N. benthamiana* and two transgenic lines were obtained. There was no difference in phenotype between the transgenic and wild type (WT) lines. Subsequently, the disease resistance of *CaSBP11* transgenic lines (lines 9 and 10) was assessed. Forty-day-old seedlings were used for this experiment. Two days after inoculation with compatible *P. capsici* (HX-9), a small area of hygrophanous lesions appeared on the detached leaves of WT plants, while the hygrophanous lesion area occupied almost half of the detached leaves of transgenic lines (Figure 7A). In addition, the average diseased area of detached leaves of transgenic lines was significantly higher than that of WT plants (Figure 7B). Subsequently, 65-days-old plants were inoculated with compatible *P. capsici* (HX-9) using the root irrigation method. Six days after inoculation with compatible *P. capsici* (HX-9), the leaves in the lower layer of WT plants turned yellow, while the leaves of transgenic lines turned yellow and the plants wilted and the junction of roots and stems appeared to exhibit constriction (Figure 7C). The expression of *CaSBP11* in transgenic plants was significantly higher than that in WT plants (Figure 7D).

In addition, the expression levels of defense-related genes (*N. benthamiana* pathogenesis-related protein PR1a, *NbPR1a*, accession number JN247448.1; *N. benthamiana* pathogenesis-related protein PR1b, *NbPR1b*, accession number XM_016587501.1; *N. benthamiana* defensin gene, *NbDEF1*, accession number X99403; *N. benthamiana* non-expressor of PR genes, *NbNPR1,* accession number AF480488) were detected after inoculation with compatible *P. capsici* (HX-9). Leaves of transgenic and WT lines were used in this experiment. Three days after inoculation with compatible *P. capsici* (HX-9), the expression levels of *NbDEF1* increased in transgenic lines compared with 0 days (Figure 7E). The expression level of *NbPR1a* in transgenic lines was higher than that in WT lines at 3 days (Figure 7E). The expression of *NbNPR1* decreased in WT plants at 3 days compared with 0 days (Figure 7E). The expression level of *NbPR1b* increased in WT plants, while it decreased in transgenic lines at 3 days compared with 0 days and was higher than that in WT plants at 0 days (Figure 7E). These results suggest that CaSBP11 plays a negative role in the defense response against *P. capsici* infection.

### 2.6. Overexpression of CaSBP11 in WT and Mutant Arabidopsis thaliana

Based on the above results, *CaSBP11* negatively regulates the expression of defense-related genes involved in the disease resistance signal pathway mediated by salicylic acid or jasmonic acid. Thus, to further study the mechanism by which *CaSBP11* involved in plant defense responses to *P. capsici* infection, the homozygous mutants for genes in the salicylic acid and jasmonic acid signaling pathways (*sid2-2*, Salk_111380; *coi1-21*, cs68754; *coi1-22*, cs68755) were randomly selected and used in this study (Supplementary Materials, Figure S3) [37,38,39]. Additionally, *NahG* (salicylate hydroxylase gene, accession number NC_007926.1), which encoded salicylate hydroxylase and prevents salicylic acid accumulation in plants, was overexpressed in *Arabidopsis thaliana* for this study. In addition, two *CaSBP11* (lines 11 and 14) and three *NahG Arabidopsis* transgenic lines (NahG-6, NahG-8 and NahG-11) were selected for the further study. The expression levels of *CaSBP11* and *NahG* in transgenic lines were significantly higher than those in WT plants (Supplementary Materials, Figure S4). Moreover, their expression levels in the *CaSBP11* and *NahG* hybrid lines (i.e., NahG/CaSBP11-8 and NahG/CaSBP11-11) were significantly higher than those of WT plants (Supplementary Materials, Figure S4). Moreover, *CaSBP11* was successfully overexpressed in *coi1-21* (*coi1-21/CaSBP11-7* and *coi1-21/CaSBP11-9*), *coi1-22* (*coi1-22/CaSBP11*) and *sid2-2* (*sid2-2/CaSBP11-2* and *sid2-2/CaSBP11-2*) lines (Supplementary Materials, Figure S4).

**Figure 8:**
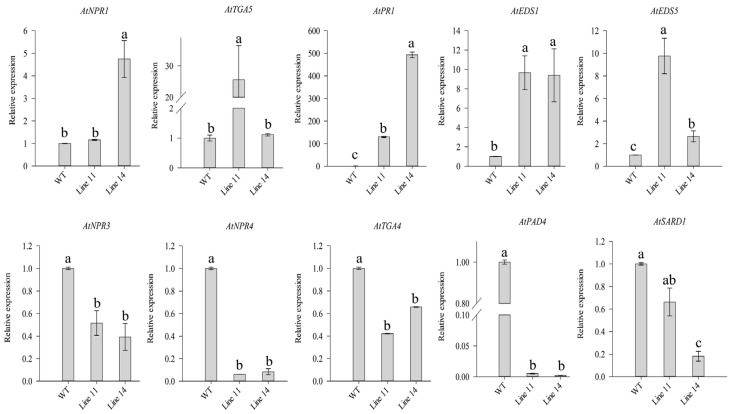
Expression of genes related to the salicylic acid signaling pathways in the *CaSBP11* transgenic lines of *Arabidopsis*. The means were analyzed using the least significant difference (LSD). Different small letters (a, ab, b, c) represent significant differences as determined by the LSD test (*p* < 0.05). Mean values and SDs for three replicates are shown.

Subsequently, the expression levels of genes related to salicylic acid signaling pathways in *CaSBP11* transgenic lines were detected. The expression levels of *AtNPR1* (nonexpresser of PR genes 1, Gene ID 842733), *AtTGA5* (TGACG motif-binding factor 5, Gene ID 830587), *AtPR1* (Pathogenesis-related gene, Gene ID 815949), *AtEDS1* (alpha/beta-Hydrolases superfamily protein, Gene ID 823964), and *AtEDS5* (MATE efflux family protein, Gene ID 830058) were increased in *CaSBP11* transgenic lines compared with those in WT lines (Figure 8). However, the expression levels of *AtNPR3* (NPR1-like protein 3, Gene ID 834545), *AtNPR4* (NPR1-like protein 4, Gene ID827710), *AtTGA4* (TGACG motif-binding factor 4, Gene ID830866), *AtPAD4* (Phytoalexin deficient 4, Gene ID 824408), and *AtSARD1* (systemic acquired resistance deficient 1, Gene ID 843716) were decreased in *CaSBP11* transgenic lines compared with those in WT lines (Figure 8). Furthermore, the expression levels of *AtNDR1* (non-race-specific disease resistance 1, Gene ID 821607) in *CaSBP11* transgenic lines were basically unchanged relative to WT lines (Supplementary Materials, Figure S5). The expression of the jasmonic acid signaling pathway related gene *AtPDF1.2* (plant defensin 1.2, Gene ID 834469) was decreased in *CaSBP11* transgenic lines compared with that in WT lines (Supplementary Materials, Figure S5)). In addition, the expression levels of ethylene signaling pathway related genes in *CaSBP11* transgenic lines were also detected. As shown in Supplementary Materials, Figure S5, the expression levels of *AtETR1* (Ethylene insensitive 1, Gene ID 842951) and *AtEIN2* (Ethylene insensitive, Gene ID 831889) were decreased in *CaSBP11* transgenic lines compared with those in WT lines.

**Figure 9:**
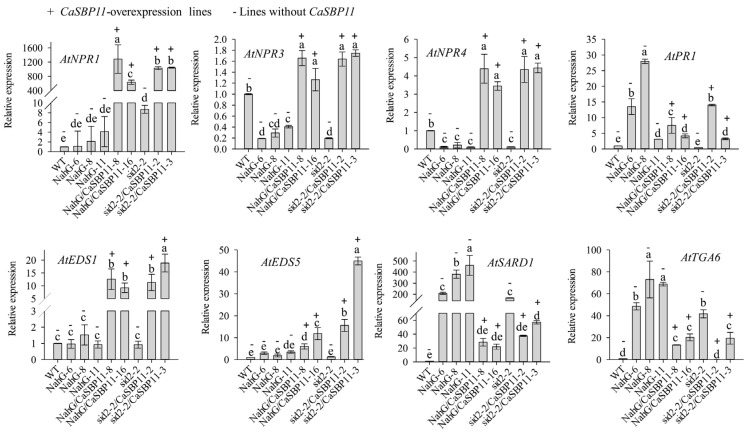
Expression of salicylic acid signaling pathway-related genes in *NahG*-overexpressing lines (NahG-6, NahG-8 and NahG-11), *NahG* and *CaSBP11* hybrid lines (NahG/CaSBP11-8 and NahG/CaSBP11-16), sid2-2 lines and *CaSBP11*-overexpressing in *sid2-2* lines (sid2-2/CaSBP11-2 and sid2-2/CaSBP11-3). The means were analyzed using the least significant difference (LSD). Different small letters (a, b, c, cd, d, de, e) represent significant differences as determined by the LSD test (*p* < 0.05). Mean values and SDs for three replicates are shown.

Additionally, the expression levels of salicylic acid signaling pathway-related genes in *NahG* overexpression lines (NahG-6, NahG-8 and NahG-11), *NahG* and *CaSBP11* hybrid lines (NahG/CaSBP11-8 and NahG/CaSBP11-16), *sid2-2* lines and *CaSBP11* overexpressing *sid2-2* lines (sid2-2/CaSBP11-2 and sid2-2/CaSBP11-3) were detected. Among the detected genes, the expression levels of *AtPR1*, *AtSARD1*, *AtTGA6* (TGACG motif-binding factor 6, Gene ID820405), *AtTGA5* and *AtMPK4* (MAP kinase 4, Gene ID 828151) were increased in *NahG*-overexpression lines compared with those in wild type lines (Figure 9, Supplementary Materials, Figure S6). However, the expression levels of *AtNPR3*, *AtNPR4* and *AtPAD4* decreased in *NahG*-overexpression lines compared with those in WT lines (Figure 9, Supplementary Materials, Figure S6). Besides, the expression levels of *AtNPR1, AtEDS1, AtEDS5* and *AtNDR1* in the *NahG*-overexpression lines was unchanged compared with those in WT lines (Figure 9, Supplementary Materials, Figure S6). Notably, the expression levels of *AtNPR1, AtNPR3*, *AtNPR4*, *AtEDS1, AtEDS5*, *AtMPK4* and *AtNDR1* in *NahG* and *CaSBP11* hybrid lines were higher than those in *NahG*-overexpression lines (Figure 9, Supplementary Materials, Figure S6). However, the expression levels of *AtPR1*, *AtSARD1, AtTGA6 and AtPAD4* in *NahG* and *CaSBP11* hybrid lines were lower than those in *NahG*-overexpression lines (Figure 9, Supplementary Materials, Figure S6). The expression levels of *AtNPR1, AtMPK4* and *AtSARD1* in *sid2-2* lines were higher than those in WT lines (Figure 9, Supplementary Materials, Figure S6). The expression levels of *AtNPR3*, *AtNPR4*, *AtPAD4 and AtNDR1* in *sid2-2* lines were lower than those in WT lines (Figure 9, Supplementary Materials, Figure S6). In addition, the expression levels of *AtPR1*, *AtEDS1*, *AtEDS5, AtTGA5 and AtTGA6* in *sid2-2* lines were unchanged compared with WT lines (Figure 9, Supplementary Materials, Figure S6). The expression levels of *AtTGA6* and *AtSARD1* in *CaSBP11*-overexpressing in *sid2-2* lines were lower than those in *sid2-2* lines (Figure 9). However, the expression levels of *AtNPR1, AtNPR3, AtNPR4, AtPR1, AtEDS1, AtEDS5, AtNDR1, AtMPK4 and AtPAD4* in *CaSBP11*-overexpressing in *sid2-2* lines were higher than those in *sid2-2* lines (Figure 9, Supplementary Materials, Figure S6). In contrast, the expression levels of *AtTGA5* in *CaSBP11*-overexpressing in *sid2-2* lines was unchanged compared with that in *sid2-2* lines (Supplementary Materials, Figure S6).

**Figure 10:**
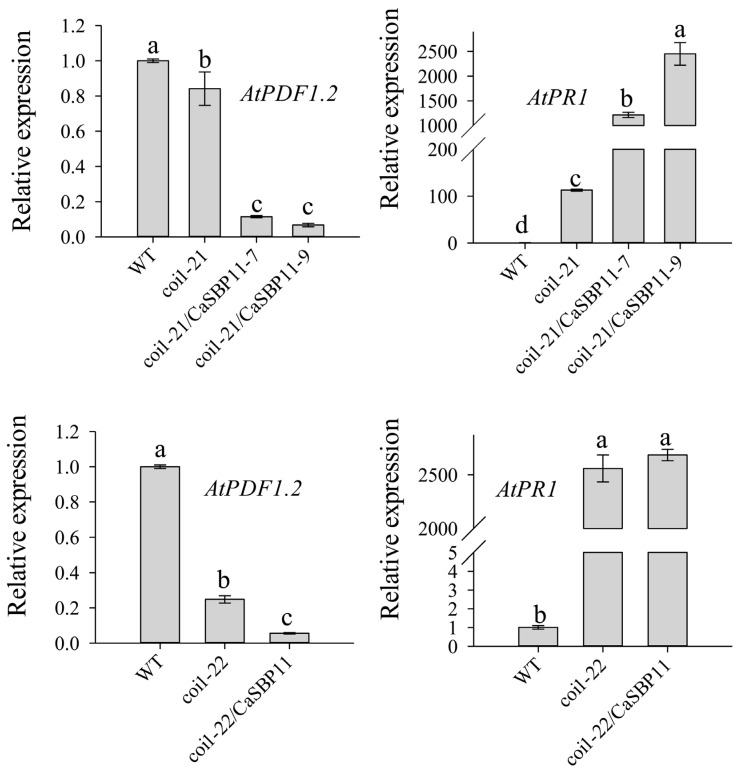
Expression levels of genes related to the jasmonic acid signal pathway in *coi1-21* and *coi1-22* lines and CaSBP11-overexpressingin *coi1-21* (coi1-21/CaSBP11-7 and coi1-21/CaSBP11-9) and *coi1-22* (coi1-22/CaSBP11). The means were analyzed using the least significant difference (LSD). Different small letters (a, b, c) represent significant differences as determined by the LSD test (*p* < 0.05). Mean values and SDs for three replicates are shown.

Furthermore, the expression levels of jasmonic acid signaling pathway-related genes in *coi1-21* and *coi1-22* lines and *CaSBP11* overexpressing in *coi1-21* (coi1-21/CaSBP11-7 and coi1-21/CaSBP11-9) and *coi1-22* lines (coi1-22/CaSBP11) were detected. As shown in Figure 10, the expression of *AtPDF1.2* in *coi1-21* and *coi1-22* lines decreased compared with that in WT lines. The expression of *AtPR1* in *coi1-21* and *coi1-22* lines was higher than that in WT lines (Figure 10). In addition, the expression level of *AtPDF1.2* in *CaSBP11*-overexpressing in *coi1-21* and *coi1-22* lines was decreased compared with that in *coi1-21* and *coi1-22* lines (Figure 10). The expression level of *AtPR1* in *CaSBP11*-overexpressing *coi1-21* and *coi1-22* lines was higher than that in *coi1-21* and *coi1-22* lines (Figure 10). These results indicated that *CaSBP11* may be involved in the salicylic acid-and jasmonic acid-mediated disease resistance signaling pathways.

## 3. Discussion

In pepper, there are 15 members of the plant-specific SBP-box gene family [18] and *CaSBP11* is one of them. The *CaSBP11* open reading frame consists of 1524 bases encoding 507 amino acids and *CaSBP11* responds to salicylic acid and methyl jasmonate treatments [18]. However, the role of *CaSBP11* in the plant defense response to *P. capsici* infection in pepper has been unclear.

*CaSBP11* has all the sequence characteristics of SBP-box family genes, namely, C3H and C2HC zinc finger structure and a nuclear localization signal [18]. CaSBP11 was observed to be localized to the nucleus (Figure 1). In addition, *CaSBP11* silencing enhanced pepper resistance to *P. capsici* infection (Figure 2). Notably, 3 days after inoculation with compatible *P. capsici*, leaves of CaSBP11-silenced and negative control plants had larger lesion areas than those of the corresponding plants inoculated with incompatible *P. capsici* (Figure 2). It has been reported that the immune system of plants mainly includes pathogen-associated molecular pattern-triggered immunity (PTI) and effector-triggered immunity (ETI) [40]. In the process of interaction between pepper plants and compatible pathogens (*Xanthomonas campestris pv*. *vesicatoria*), when the effectors of bacteria cannot be recognized by disease resistance proteins, PTI will be activated along with the expression of PR proteins. Subsequently, the basic defense response of plants was established through the salicylic acid-mediated signaling pathway [41]. Furthermore, when the expression of PR protein cannot exceed the critical point of a hypersensitive response, a plant becomes diseased [41]. In the process of interactions between pepper plants and incompatible pathogens (*Xanthomonas campestris pv*. *vesicatoria*), bacterial effectors can be recognized by disease resistant proteins and initiate effector induced immune responses in plants [41]. The occurrence of ETI immune response can accelerate and enhance the occurrence of PTI immune response, which leads to an increase in plant disease resistance [40]. However, in the process of natural selection, the diversity of recognition effector genes and the increase of effector genes can lead to pathogens evading effector- induced immune responses [40]. In compatible plant-microbe interactions, susceptible cell death occurs relatively late in the course of infection [40]. This may be the reason why the diseased areas of detached leaves after inoculation with compatible *P. capsici* were larger than those inoculated with incompatible *P. capsici,* though both of them were infected.

It has been reported that *CaSAR8.2* is a marker gene responding to pathogen infection and involved in the salicylic acid-mediated disease resistance signaling pathway [42]. *CaBPR1* is involved in the hypersensitive response in pepper and can be induced by *Xanthomonas campestris pv. vesicatoria* [43]. *CaDEF1* is related to the jasmonic acid-mediated signal transduction pathway and responds to plant infection and other environmental stresses [44]. *CaPO1* regulates the level of hydrogen peroxide and the activity of peroxidase during the hypersensitive response induced by the interactions between pepper and incompatible pathogens [45]. Therefore, the expression levels of these genes in CaSBP11-silenced plants and negative control plants were detected after inoculation with compatible and incompatible *P. capsici*. As shown in Figure 4 and Figure 5, the expression levels of these defense-related genes increased in the CaSBP11-silenced plants after inoculation with compatible and incompatible *P. capsici* and were higher than that in negative control plants at 2 days. In addition, the expression levels of these genes (except *CaSAR8.2*) after inoculation with incompatible *P. capsici* were much higher than that after inoculation with compatible *P. capsici*.

It has been reported that PR genes, including *CaPR10*, *CaBPR1 and CaPOA1*, are regulated by salicylic acid-mediated signaling pathways [41]. In addition, *CaHIR1* plays a negative role in the interaction between pepper and compatible and incompatible pathogens (*Xanthomonas campestris pv. vesicatoria*) and the expression levels of *CaPR10*, *CaBPR1*, *CaPOA1 and CaDEF1* were significantly induced in the *CaHIR1* silenced plants [41]. The expression of defense related genes in pepper plants was positively correlated with plant resistance [46]. It should be noted that the expression levels of *CaPO1*, *CaSAR8.2*, *CaBPR1 and CaDEF1* in CaSBP11-silenced plants were significantly higher than those in the negative control plants at 0 days (Figure 4 and Figure 5). Therefore, we speculated that *CaSBP11* may enhance pepper resistance to *P. capsici* infection by regulating the expression of these defense-related genes.

To test this hypothesis, *CaSBP11* was transiently expressed in pepper. The expression levels of these defense-related genes in *CaSBP11* transient expression lines were then detected. The expression levels of *CaPO1*, *CaSAR8.2*, *CaBPR1 and CaDEF1* were significantly inhibited in the transient *CaSBP11* expression lines (Figure 6). These results suggest that *CaSBP11* may be involved in the defense response of pepper to *P. capsici* infection by regulating the expression of defense-related genes. However, it is unclear whether *CaSBP11* is involved in the signal pathways of these defense-related genes.

Subsequently, the function of *CaSBP11* in the plants defense response against *P. capsici* infection was further verified in *N. benthamiana*. Overexpression of *CaSBP11* in *N. benthamiana* increased the sensitivity of transgenic lines to *P. capsici* infection (Figure 7). Furthermore, *NbPR1a* expression was induced in transgenic lines at 3 days after inoculation with *P. capsici*. In addition, the expression level of *NbPR1b* in the transgenic lines was higher than that of WT lines at 0 days (Figure 7). It has been previously reported that *NbPR1a* and *NbPR1b* can be significantly induced by tobacco mosaic virus (TMV) in tobacco [47]. *NbPR1a* is a marker gene for the salicylic acid signaling pathway, which is related to systemic acquired resistance of plants and participates in the plant defense response to *Pseudomonas syringae* infection in tobacco [48]. *PR1b* is a jasmonate-responsive gene in tobacco [49]. In addition, the overexpression of *CaC3H14* in tobacco enhanced the resistance of transgenic lines to *Ralstonia solanacearum* infection and induced the expression of *PR1b* in transgenic lines and WT plants. However, *PR1b* expression in transgenic plants was significantly lower than that in WT plants [50].

**Figure 11:**
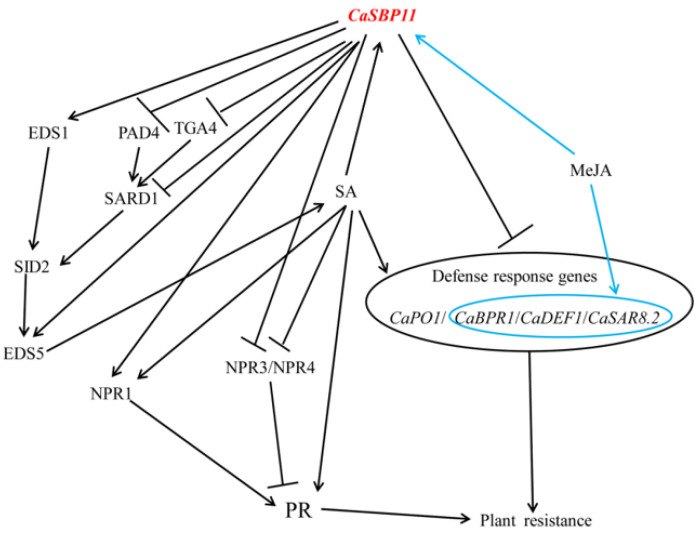
Proposed model for *CaSBP11* participating in plant defense response. Arrows indicate positive regulation, while those without arrows indicate negative regulation. The genes that are not marked Ca are expected to exist in pepper as homologues of those in *Arabidopsis*. *CaPO1*: Pepper peroxidase-like gene; *CaBPR1*: Pepper pathogenesis-related (PR)-1 protein; *CaDEF1:* Pepper defensin gene; *CaSAR8.2*: Systemic acquired resistance gene; *EDS1*: alpha/beta-Hydrolases superfamily protein; *PAD4*: Phytoalexin deficient 4; *TGA4*: TGACG motif-binding factor 4; *SARD1*: systemic acquired resistance deficient 1; *SID2-2*: Salicylic acid induction deficient 2; *EDS5*: MATE efflux family protein; *NPR1*: non-expressor of PR genes 1; *NPR3*: NPR1-like protein 3; *NPR4*:NPR1-like protein 4; *PR*: Pathogenesis-related gene.

It has been reported that *SPL9* interacts with JAZ protein in *Arabidopsis thaliana* and negatively regulates jasmonic acid signal pathway by promoting the accumulation of *JAZ3* [33]. Similarly, *VpSBP5* may participate in the plant defense response against powdery mildew infection through salicylic acid-and jasmonic acid-mediated signal transduction pathways in grapes [36]. Therefore, to further assess whether *CaSBP11* is involved in the salicylic acid-and jasmonic acid-mediated signal pathways. *CaSBP11* was overexpressed in *Arabidopsis thaliana,* both in WT plants and salicylic acid synthesis pathway (*sid2-2*) and jasmonic acid synthesis pathway (*coi1-21* and *coi1-22*) mutants. In addition, *CaSBP11* was co-expressed in *Arabidopsis thaliana* with *NahG*, which can prevent salicylic acid accumulation. As shown in Figure 8, Figure 9 and Figure 10, *CaSBP11* can regulate the expression of genes in the salicylic acid and jasmonic acid signaling pathways to different degrees. Among the salicylic acid-mediated signaling pathway genes, *NDR1*, *EDS1*, *PAD4*, *SID2*, *EDS5 and NPR1* play a positive regulatory role in *Arabidopsis* defense against *P. capsici* infection [51]. These genes, except *NPR1*, are upstream of the salicylic acid signaling pathway (Supplementary Materials, Figure S7). However, overexpression of *NahG* in *Arabidopsis thaliana* appeared to inhibit the synthesis of salicylic acid and change the expression of related genes in the salicylic acid signaling pathway (Figure 10, Supplementary Materials, Figure S7) [51]. *CBP60g* and *SARD1* are located between *PAD4* and *SID2* (Supplementary Materials, Figure S7) [52] and promote the production of salicylic acid when *Arabidopsis* recognizes microbial-related molecular patterns and there is functional redundancy between CBP60g and SARD1 [52]. However, CBP60g can inhibit bacterial growth by binding to calmodulin, while SARD1 does not need to bind calmodulin [52]. In addition, CBP60g has a greater function in the early stage of plant defense responses, while SARD1 has a greater function in the later stage of defense responses [52]. The transcription factor TGA1/4 can regulate the synthesis of salicylic acid by regulating the expression of *CBP60g* and *SARD1* [53] (Supplementary Materials, Figure S7). Salicylic acid can promote the expression of *NPR1*, which can interact with TGA2/5/6 gene to regulate PR gene expression [54]. TGA2/5/6 have redundant gene functions, which are indispensable in plant acquired resistance and negatively regulate the expression of PR genes [55]. Salicylic acid inhibited the expression of *NPR3* and *NPR4* (Supplementary Materials, Figure S7) [54,56]. In addition, gene functional redundancy exists between *NPR3* and *NPR4*, in contrast with *NPR1*, *NPR3 and NPR4* also interact with TGA2/5/6 (Supplementary Materials, Figure S7) [54]. *MPK4* negatively regulates plant acquired resistance and plays a negative role in the plant defense response against *P. capsici* infection in *Arabidopsis thaliana* (Supplementary Materials, Figure S7) [51,57]. Moreover, *MPK4* can regulate salicylic acid and jasmonic acid signal-mediated defense responses by regulating the expression of *PAD4* and *EDS1* [58]. However, jasmonic acid receptor (COI) mutations, made plants insensitive to jasmonic acid and the jasmonic acid signaling pathway was thus blocked [38,39]. In addition, JAR1 and PDF1.2 in the jasmonic acid signaling pathway play a positive role in plant defense responses to *P. capsici* infection in *Arabidopsis thaliana* (Supplementary Materials, Figure S7) [51]. However, CaSBP11 can regulate the expression of these genes in the salicylic acid and jasmonic acid signal pathways to different degrees. This indicates that CaSBP11 can participate in the signaling pathways mediated by salicylic acid and jasmonic acid by regulating the expression of genes related to the salicylic acid and jasmonic acid signal pathways. Thus, based on the above results, we speculate that CaSBP11 participates in plant defense responses by regulating the expression of defense-related genes on the one hand (Figure 11). On the other hand, by inhibiting the expression of genes upstream of the salicylic acid signaling pathway (e.g., *PAD4*, *TGA4 and SARD1)*, thereby inhibiting the production of salicylic acid, the expression of PR genes and their participation in the defense response of plants is affected (Figure 11). However, at the same time, CaSBP11 can promote the *NPR1* expression and inhibit the *NPR3* and *NPR4* expression (Figure 11). NPR1 can interact with TGA2/5/6 to further promote PR gene expression, while NPR3 and NPR4 can interact with TGA2/5/6 to inhibit PR gene expression. In addition, CaSBP11 can also promote the expression of EDS1 and EDS5, which in turn promote the synthesis of salicylic acid (Figure 11). Thus, how the CaSBP11 regulates the defense response of plants merits further research.

## 4. Materials and Methods

### 4.1. Plant Material and Pathogen Preparation

Pepper (*Capsicum annuum*) cultivar AA3 and *P. capsici* strain PC (incompatible.) and HX-9 (compatible) were obtained from the Vegetable Biotechnology and Germplasm Resources Innovation Laboratory, College of Horticulture, Northwest A&F University, Yangling, P. R. China. Both Columbia-0 ecotype *Arabidopsis thaliana* (Col-0) and *N. benthamiana* were obtained from propagation within the laboratory. The *Arabidopsis sid2-2* mutant (Salk_111380) and *coi1-21* (cs68754) and *coi1-22* (cs68755) mutants were derived from the SALK mutant library. Plants were grown in a growth chamber at 22/18 °C (day/night temperatures) with a 16 h photoperiod. *P. capsici* was cultured in the dark at 28 °C on potato glucose agar medium (PDA),which includes potato, glucose and agar (200 g potato, 20 g glucose and 17 g agar per 1000 mL). Sporulation induction and spore release were performed using a modification of the method described by Wang et al. [59]. Briefly, *P. capsici* was first cultured on PDA at 28 °C for 5 days. Then, the cultivated *P. capsici* was divided into 8-mm diameter discs. After that, ten discs were cultured in the dark for 3 days in 90-mm-diameter Petri dishes with 15–20 mL of 2% (*w/v*) cleared carrot broth at 28 °C. The 2% (*w/v*) cleared carrot broth was prepared as follows: 200 g of carrot was first cut into small pieces, added to 1 L distilled water and boiled for 30 min; the resulting solution was filtered through three layers of non-woven fabric; the filtered liquid was sterilized at 121 °C for 21 min and used after cooling. The cultures were then washed twice with sterile distilled water and covered with 15–20 mL of Petri broth (KH_2_PO_4_, 0.15 g; Ca(NO_3_)_2_, 0.4 g; CaCl_2_, 0.06 g; Mg(NO_3_)_2_, 0.15 g; each per 1000 mL). These cultures were further incubated at 28°C for five more days. The cultures were then chilled for 30 min at 4 °C to induce zoospore release and then incubated for 1 h at room temperature. The zoospore concentration was adjusted to 1 × 10^5^ spores/mL using a hemocytometer according to the method described by Jin et al. [60]. Then, 5 mL of this zoospore culture was used to inoculate the *CaSBP11* silenced and transgenic *N. benthamiana* plants following the root-drench method described by Wang [61]. The detached leaf inoculation was performed according to the method described by Zhang et al. [62].

### 4.2. Subcellular Localization of CaSBP11Protein in Pepper

The coding region of *CaSBP11* without its termination codon was amplified and cloned into a pVBG2307: GFP vector (which contains a CaMV35S promoter that comes from PBI121 [63] and a GFP gene) between the *BamH*I and *Sma*I restriction sites to yield the final pVBG2307:CaSBP11:GFP plasmid (Supplementary Materials, Table S1) The recombinant fusion pVBG2307:CaSBP11:GFP plasmid was confirmed by sequencing performed by Sangon-Biotech Company (Shanghai, China). Then, the recombined vector (pVBG2307:CaSBP11:GFP) was transformed into the *Agrobacterium tumefaciens* strain GV3101 via the freeze-thaw method. Next, GV3101 cells carrying the pVBG2307:CaSBP11:GFP (CaMV35S:CaSBP11:GFP) and pVBG2307:GFP (CaMV35S:GFP) constructs were cultured overnight in Luria-Bertani (LB) medium with the appropriate antibiotics. Then, a prepared buffer (10 mM MES, pH 5.7, 10 mM MgCl2 and 200 μm acetosyringone) was used to create a cell suspension. The cell suspension (OD = 0.8) was then injected into the leaves of *N. benthamiana* with a needless syringe [64]. After injection, the plants were first cultured in darkness at 22 °C for 12 h and then cultured at 22/18 °C (day/night temperature) with a 16-h photoperiod for two days. After that, a fluorescent confocal microscope (Olympus, Tokyo, Japan) with a 488 nm excitation wavelength was used to detect green fluorescence.

### 4.3. Virus Induced Gene Silencing (VIGS) of CaSBP11 in Pepper

For the VIGS assay, a specific fragment (224bp) from the *CaSBP11* gene in pepper was amplified using specific primers (Supplementary Materials, Table S2). The specificity of these primers was assessed using NCBI Primer BLAST (http://www.ncbi.nlm.nih.gov/tools/primer-blast/index.cgi?LINK_LOC=blasthome) and pepper database (http://peppergenome.snu.ac.kr/). Then, the obtained product was cloned into the TRV2 vector using the double digestion method with *BamH*I and *Kpn*I enzymes (Trans Gen Biotech, Beijing, China). Then, the recombinant fusion TRV2:*CaSBP11* plasmid was confirmed by sequencing conducted by Sangon-Biotech Company (Shanghai, China). The recombined vector, TRV2 (negative control), TRV2:*CaPDS* (phytoenedesaturase, positive control) and TRV1 were transformed into *Agrobacterium tumefaciens* strain GV3101 via the freeze-thaw method. Pepper seedlings at the two-true leaf stage (40 days after sowing) were subjected to *CaSBP11* silencing as described by Zhang et al. [65]. All the injected plants were grown in a growth chamber following the conditions described by Wang [61]. Forty-five days after infection, root and leaf samples from the silenced and control plants were collected for measurement of silencing efficiency. Then, the assay of the detached leaves was conducted as described by Zhang et al. [62]. Next, 5 mL of 1 × 10^5^ spores/mL zoospore solution of *P. capsici* was used to inoculate the silenced and control plants, respectively, using the root-drench method [61]. Then, roots from the silenced and control plants were collected and stored at −80 °C.

### 4.4. Transient Expression of CaSBP11 in Pepper

The recombined vector (pVBG2307:CaSBP11:GFP) was used for transient expression in pepper. Gv3101 cells carrying pVBG2307:CaSBP11:GFP vector and pVBG2307:GFP vector (used as control) were cultured overnight in LB medium with the appropriate antibiotics. Then, a prepared buffer (10 mM MES, pH 5.7, 10 mM MgCl2 and 200 μm acetosyringone) was to create cell suspensions. The cell suspensions (OD = 0.8) were then injected into the leaves of pepper plants bearing 6–8 true leaves with a needless syringe [64]. After injection, the pepper plants were first cultured in darkness at 25 °C for 12 h and then grown at 22/18 °C (day/night temperatures) with a 16 h photoperiod at 70% relative humidity. After 2 days of cultivation under the above conditions, leaves of pepper plants were collected and stored at –80 °C.

### 4.5. Overexpression of CaSBP11 in N. benthamiana

The recombined vector (pVBG2307:CaSBP11:GFP) was used for *CaSBP11* overexpressionin *N. benthamiana.* In this study, *CaSBP11* transgenic lines of *N. benthamiana* were obtained by *Agrobacterium tumefaciens*-mediated leaf disc transformation [66]. Two kanamycin resistant *CaSBP11* transgenic lines were obtained, with RNA confirmation in both transformed lines. T1 generation seeds were obtained from the regenerated T0 generation plants and T2 generation seeds were obtained from T1 generation plants. In this experiment, T3 generation plants were selected for further study.

### 4.6. Overexpression of CaSBP11 in WT and Mutant Arabidopsis thaliana

The recombined vector (pVBG2307:CaSBP11:GFP) was used for *CaSBP11* overexpression in WT and mutant *Arabidopsis thaliana*. Thus, *CaSBP11* was over-expressed in WT *Arabidopsis thaliana* and the *Arabidopsis thaliana* mutants *sid2-2* (Salk_111380), *coi1-21* (cs68754) and *coi1-22* (cs68755), respectively. The transgenic lines with kanamycin resistance were obtained from the *Arabidopsis thaliana sid2-2*, *coi1-21 and coi1-22* mutants. In addition, the salicylate hydroxylase gene (*NahG*) was cloned from *Pseudomonas putida* ND6 and successfully cloned into the pVBG2307 vector [67,68]. Subsequently, *NahG* was successfully over-expressed in *Arabidopsis thaliana* by the floral dip method. Then, the obtained homozygous *NahG* transgenic line was used as the female parent and the *CaSBP11 Arabidopsis thaliana* transgenic line was used as the male parent in crosses. Finally, transgenic lines containing *NahG* and *CaSBP11* were obtained. Primers used in vector construction and mutation detection of *sid2-2*, *coi1-21* and *coi1-22* are described in Supplementary Materials, Table S1.

### 4.7. Disease Index Percentage Statistics

The plant disease index percentage statistics refer to the method described by Zhang [69]. In this experiment, 16 days after inoculation with *P. capsici*, the plant disease phenotype was categorized into five levels for the CaSBP11-silenced and control plants. The specific disease levels were as follows: level 0, no symptoms; level 1, the lower leaves of the plant turned yellow or wilted; level 2, the lower leaves of the plant have an obvious deciduous phenomenon or the whole plant has wilted; level 3, blackening of stem base and all leaves have fallen off except at growth points; level 4, the whole plant is dead. The disease index percentage was calculated using the Equation (1) [70]:

Disease index percentage = [(∑the numerical grade of disease×number of disease plants of this grade)/(the highest grade of disease×total number of surveys)] × 100(1)

### 4.8. RNA Extraction and Quantitative Real-Time PCR

RNA was extracted according to the method described by Guo et al. [71]. Then, cDNAs were synthesized using the instructions for the PrimeScript Kit (Takara, Dalian, China). The cDNA concentration was then diluted to 50 ng/μL for quantitative real-time PCR (qRT-PCR), which was performed on the iCycleriQ^TM^ Multicolor PCR Detection System (Bio-Rad, Hercules, CA, USA) according to the following thermal cycling procedure: pre-denaturation at 95 °C for 1 min, followed by 40 cycles of denaturation at 95 °C for 10 s, annealing at 56 °C for 30 s and extension for 30 s at 72 °C. The primers used for qRT-PCR are shown in Supplementary Materials, Table S2. The specificity of all primers was assessed using NCBI (http://www.ncbi.nlm.nih.gov/tools/primer-blast/index.cgi?LINK_LOC=blasthome). Gene expression was quantified and normalized to that of actin (*CaActin2*, accession number AY572427; *Nbactin-97*, accession number LOC109206422; *Atactin2*, accession number NC_003074) expression [72,73,74,75].

### 4.9. Statistical Analysis

In this study, Data Processing System 7.05 (DPS 7.05, China) with comprehensive experimental design and statistical analysis functions was used for data analysis. Following an analysis of variance (ANOVA), the least significant difference (LSD) test was used to access the significance of differences (*p <* 0.01 or *p <* 0.05). All experiments were performed and analyzed separately with at least three biological replicates.

## 5. Conclusions

CaSBP11 protein was confirmed to be localized to the nucleus. Silencing of *CaSBP11* enhanced plant defense responses against *P. capsici* infection. The expression of defense-related genes (*CaPO1*, *CaBPR1*, *CaDEF1* and *CaSAR8.2*) was increased in *CaSBP11*-silenced plants. However, these genes were inhibited in the *CaSBP11* transient expression plants. Furthermore, over-expression of *CaSBP11* in *N. benthamiana* increased its susceptibility to *P. capsici* infection. Overexpression of *CaSBP11* in *Arabidopsis thaliana* and *sid2-2*, *coi1-21 and coi1-22* mutants appeared to regulate the expression of genes in the salicylic acid and jasmonic acid signaling pathways to different degrees. These results indicate that *CaSBP11* plays a negative role in plant defense responses to *P. capsici* infection. Moreover, *CaSBP11* may participate in the defense response of pepper plants to *P. capsici* infection by regulating the expression of defense-related genes and may also be involved in the salicylic acid-and jasmonic acid-mediated disease resistance signaling pathways.

## Supplementary Materials

The following are available online at https://www.mdpi.com/1422-0067/21/23/9065/s1, Figure S1: Phenotype and silencing efficiency of CaSBP11-silenced plant, Figure S2: The symptom-based scale used for the disease of CaSBP11-silenced and control plants category, Figure S3: Screening of *sid2-2*, *coi1-21* and *coi1-22* homozygous lines, Figure S4: Overexpression of *CaSBP11* in *Arabidopsis* and *NahG*-overexpression lines, *sid2-2*, *coi1-21,* and *coi1-22* lines, Figure S5: Expression of the salicylic acid, jasmonate and ethylene signal pathways genes in *CaSBP11* overexpressing lines of *Arabidopsis thaliana,* Figure S6: Expression of the salicylic acid signaling pathway-related genes in *NahG*-overexpressing lines (NahG-6, NahG-8, and NahG-11 ), *NahG* and *CaSBP11* hybrid lines (NahG/CaSBP11-8 and NahG/CaSBP11-16), *sid2-2* lines and *CaSBP11* overexpression in *sid2-2* lines (sid2-2/CaSBP11-2 and sid2-2/CaSBP11-2), Figure S7: Partial pattern of salicylic acid and jasmonate signalling related gene in *Arabidopsis* [38,39,51,52,53,54,55,56,57,58], Table S1: Primers names and their sequences used for vector construction and mutation detection in this study, Table S2: Primers names and their sequences used in this study for quantitative PCR.

## References

[1] Barchenger D.W., Lamour K.H., Bosland P.W. (2018). Challenges and strategies for breeding resistance in *Capsicum annuum* to the multifarious pathogen. *Phytophthora capsici*. Front. Plant Sci..

[2] Liu K.K. (2009). Studies on the Resistance and Its Mechanisms of Pepper to Phytophthora Capsici.

[3] Hausbeck M.K., Lamour K.H. (2004). *Phytophthora capsici* on vegetable crops: Research progress and management challenges. Plant Dis..

[4] Granke L.L., Quesada-ocampo L., Hausbeck M.K. (2012). Advances in research on *Phytophthora capsici* on vegetable crops in the United States. Plant Dis..

[5] He Y.M., Luo D.X., Khan A., Liu K.K., Arisha M.H., Zhang H.X., Cheng G.X., Ma X., Gong Z.H. (2018). *CanTF*, a novel transcription factor in pepper, is involved in resistance to *Phytophthora capsici* as well as abiotic stresses. Plant Mol. Biol. Rep..

[6] Ng D.W.K., Abeysinghe J.K., Kamali M. (2018). Regulating the regulators: The control of transcription factors in plant defense signaling. Int. J. Mol. Sci..

[7] Mao G., Meng X., Liu Y., Zheng Z., Chen Z., Zhang S. (2011). Phosphorylation of a WRKY transcription factor by two pathogen-responsive MAPKs drives phytoalexin biosynthesis in *Arabidopsis*. Plant Cell.

[8] Chen C., Chen Z. (2002). Potentiation of developmentally regulated plant defense response by *AtWRKY18*, a pathogen-induced *Arabidopsis* transcription factor. Plant Physiol..

[9] Jin J., Zhang H., Ali M., Wei A., Luo D., Gong Z. (2019). The *CaAP2/ERF064* regulates dual functions in pepper: Plant cell death and resistance to *Phytophthora capsici*. Genes.

[10] Klein J., Saedler H., Huijser P. (1996). A new family of DNA binding proteins includes putative transcriptional regulators of the *Antirrhinum majus* floral meristem identity gene SQUAMOSA. Mol. Gen. Genet..

[11] Yamasaki K., Kigawa T., Inoue M., Tateno M., Yamasaki T., Yabuki T., Aoki M., Seki E., Matsuda T., Nunokawa E. (2004). A novel zinc-binding motif revealed by solution structures of DNA-binding domains of *Arabidopsis* SBP-family transcription factors. J. Mol. Biol..

[12] Cardon G., Höhmann S., Klein J., Nettesheim K., Saedler H., Huijser P. (1999). Molecular characterisation of the *Arabidopsis* SBP-box genes. Gene.

[13] Lännenpää M., Jänönen I., Hölttä-Vuori M., Gardemeister M., Porali I., Sopanen T. (2004). A new SBP-box gene *BpSPL1* in silver birch (*Betula pendula*). Physiol. Plant.

[14] Poole M., Hong Y., Thompson A.J., King G.J., Manning K., To M., Giovannoni J.J., Seymour G.B. (2006). A naturally occurring epigenetic mutation in a gene encoding an SBP-box transcription factor inhibits tomato fruit ripening. Nat Genet..

[15] Li J., Hou H., Li X., Xiang J., Yin X., Gao H., Zheng Y., Bassett C.L., Wang X. (2013). Genome-wide identification and analysis of the SBP-box family genes in apple (Malus×domestica Borkh.). Plant Physiol. Biochem..

[16] Zhang L., Wu B., Zhao D., Li C., Shao F., Lu S. (2014). Genome-wide analysis and molecular dissection of the SPL gene family in *Salvia miltiorrhiza*. J. Integr. Plant Biol..

[17] Zhang X., Dou L., Pang C., Song M., Wei H., Fan S., Wang C., Yu S. (2015). Genomic organization, differential expression, and functional analysis of the SPL gene family in *Gossypium hirsutum*. Mol. Genet Genom..

[18] Zhang H.-X., Jin J.-H., He Y.-M., Lu B.-Y., Li D.-W., Chai W.-G., Khan A., Gong Z.-H. (2016). Genome-wide identification and analysis of the SBP-Box family genes under *Phytophthora capsici* stress in pepper (*Capsicum annuum* L.). Front. Plant Sci..

[19] Song S., Zhou H., Sheng S., Cao M., Li Y., Pang X. (2017). Genome-wide organization and expression profiling of the SBP-box gene family in Chinese jujube (*Ziziphus jujuba* mill.). Int. J. Mol. Sci..

[20] Zhou Q., Zhang S., Chen F., Liu B., Wu L., Li F., Zhang J., Bao M., Liu G. (2018). Genome-wide identification and characterization of the SBP-box gene family in *Petunia*. BMC Genom..

[21] Li J., Gao X., Sang S., Liu C. (2019). Genome-wide identification, phylogeny, and expression analysis of the SBP-box gene family in *Euphorbiaceae*. BMC Genom..

[22] Hou H.M., Li J., Gao M., Singer S.D., Wang H., Mao L.Y., Fei Z.J., Wang X.P. (2013). Genomic organization, phylogenetic comparison and differential expression of the SBP-box family genes in grape. PLoS ONE.

[23] Zhang S.D., Ling L.Z. (2014). Genome-wide identification and evolutionary analysis of the SBP-box gene family in castor bean. PLoS ONE.

[24] Ma Y., Guo J.W., Bade R., Men Z.H., Hasi A. (2014). Genome-wide identification and phylogenetic analysis of the SBP-box gene family in melons. Genet. Mol. Res..

[25] Xu Z., Sun L., Zhou Y., Yang W., Cheng T., Wang J., Zhang Q. (2015). Identification and expression analysis of the SQUAMOSA promoter-binding protein (SBP)-box gene family in *Prunus mume*. Mol. Genet. Genom..

[26] Wu Z., Cao Y., Yang R., Qi T., Hang Y., Lin H., Zhou G., Wang Z.Y., Fu C. (2016). Switchgrass SBP-box transcription factors *PvSPL1* and *2* function redundantly to initiate side tillers and affect biomass yield of energy crop. Biotechnol. Biofuels.

[27] Zhang D., Han Z., Li J., Qin H., Zhou L., Wang Y., Zhu X., Ma Y., Fang W. (2019). Genome-wide analysis of the SBP-box gene family transcription factors and their responses to abiotic stresses in tea (*Camellia sinensis*). Genomics.

[28] Unte U.S., Sorensen A., Pesaresi P., Gandikota M., Leister D., Saedler H., Huijser P. (2003). *SPL8*, an SBP-Box gene that affects pollen sac development in *Arabidopsis*. Plant Cell.

[29] Zhang B., Xu W., Liu X., Mao X., Li A., Wang J., Chang X., Zhang X., Jing R. (2017). Functional conservation and divergence among homoeologs of *TaSPL20* and *TaSPL21*, two SBP-Box genes governing yield-related traits in Hexaploid Wheat. Plant Physiol..

[30] Gao R., Gruber M.Y., Amyot L., Hannoufa A. (2018). *SPL13* regulates shoot branching and flowering time in *Medicago sativa*. Plant Mol. Biol..

[31] Stone J.M., Liang X., Nekl E.R., Stiers J.J. (2005). *Arabidopsis AtSPL14*, a plant-specific SBP-domain transcription factor, participates in plant development and sensitivity to fumonisin B1. Plant J..

[32] Padmanabhan M.S., Ma S., Burch-Smith T.M., Czymmek K., Huijser P., Dinesh-Kumar S.P. (2013). Novel positive regulatory role for the *SPL6* transcription factor in the N TIR-NB-LRR receptor-mediated plant innate immunity. PLoS Pathog..

[33] Mao Y.B., Liu Y.Q., Chen D.Y., Chen F.Y., Fang X., Hong G.J., Wang L.J., Wang J.W., Chen X.Y. (2017). Jasmonate response decay and defense metabolite accumulation contributes to age-regulated dynamics of plant insect resistance. Nat. Commun..

[34] Jung C., Yeu S.Y., Koo Y.J., Kim M., Choi Y.D., Cheong J.J. (2007). Transcript profile of transgenic *Arabidopsis* constitutively producing methyl jasmonate. J. Plant Biol..

[35] Yao S., Yang Z., Yang R., Huang Y., Guo G., Kong X., Lan Y., Zhou T., Wang H., Wang W. (2019). Transcriptional regulation of miR528 by *OsSPL9* orchestrates antiviral response in rice. Mol. Plant.

[36] Hou H., Yan Q., Wang X., Xu H. (2013). A SBP-Box gene *VpSBP5* from chinese wild vitis species responds to *Erysiphe necator* and defense signaling molecules. Plant Mol. Biol. Rep..

[37] Alonso J.M., Stepanova A.N., Leisse T.J., Kim C.J., Chen H., Shinn P., Stevenson D.K., Zimmerman J., Barajas P., Cheuk R. (2003). Genome-wide insertional mutagenesis of *Arabidopsis* thaliana. Science.

[38] He Y., Chung E.H., Hubert D.A., Tornero P., Dangl J.L. (2012). Specific missense alleles of the *Arabidopsis* jasmonic acid co-receptor COI1 regulate innate immune receptor accumulation and function. PLoS Genet..

[39] Hubert D.A., He Y., McNulty B.C., Tornero P., Dangl J.L. (2009). Specific *Arabidopsis* HSP90.2 alleles recapitulate RAR1 cochaperone function in plant NB-LRR disease resistance protein regulation. Proc. Natl. Acad. Sci. USA.

[40] Jones J.D.G., Dangl J.L. (2006). The plant immune system. Nature.

[41] Choi H.W., Kim Y.J., Hwang B.K. (2011). The hypersensitive induced reaction and leucine-rich repeat proteins regulate plant cell death associated with disease and plant immunity. Mol. Plant Microbe Interact..

[42] Lee S.C., Hwang B.K. (2003). Identification of the pepper *SAR8.2* gene as a molecular marker for pathogen infection, abiotic elicitors and environmental stresses in *Capsicum annuum*. Planta.

[43] Kim Y.J., Hwang B.K. (2000). Pepper gene encoding a basic pathogenesis-related 1 protein is pathogen and ethylene inducible. Physiol. Plant.

[44] Do H.M., Lee S.C., Jung H.W., Sohn K.H., Hwang B.K. (2004). Differential expression and in situ localization of a pepper defensin (*CADEF1*) gene in response to pathogen infection, abiotic elicitors and environmental stresses in *Capsicum annuum*. Plant Sci..

[45] Do H.M., Hong J.K., Jung H.W., Kim S.H., Ham J.H., Hwang B.K. (2003). Expression of peroxidase-like genes, H2O2 production, and peroxidase activity during the hypersensitive response to *Xanthomonas campestris* pv. *vesicatoria* in *Capsicum annuum*. Mol. Plant Microbe Interact..

[46] Zhang Y.L. (2013). Defence Responses of Pepper (Capsicum Annuum L.) and Grafting Seedings to Phytophthora Capsici Leonia and the Function Analysis of CaRGA2 Gene.

[47] van Huijsduijnen R.A.M.H., Cornelissen B.J.C., van Loon L.C., van Boom J.H., Tromp M., Bol J.F. (1985). Virus-induced synthesis of messenger RNAs for precursors of pathogenesis-related proteins in tobacco. EMBO J..

[48] Cheol Song G., Sim H.-J., Kim S.-G., Ryu C.-M. (2016). Root-mediated signal transmission of systemic acquired resistance against above-ground and below-ground pathogens. Ann. Bot..

[49] Sohn S.I., Kim Y.H., Kim B.R., Lee S.Y., Lim C.K., Hur J.H., Lee J.Y. (2007). Transgenic tobacco expressing the *hrpNEP* gene from Erwinia pyrifoliae triggers defense responses against *Botrytis cinerea*. Mol. Cells.

[50] Qiu A., Lei Y., Yang S., Wu J., Li J., Bao B., Cai Y., Wang S., Lin J., Wang Y. (2018). *CaC3H14* encoding a tandem CCCH zinc finger protein is directly targeted by *CaWRKY40* and positively regulates the response of pepper to inoculation by *Ralstonia solanacearum*. Mol. Plant Pathol..

[51] Wang Y.A.N., Bouwmeester K., Van de Mortel J.E., Shan W., Govers F. (2013). A novel *Arabidopsis*–oomycete pathosystem: Differential interactions with *Phytophthora capsici* reveal a role for camalexin, indole glucosinolates and salicylic acid in defence. Plant Cell Environ..

[52] Wang L., Tsuda K., Truman W., Sato M., Nguyen L.V., Katagiri F., Glazebrook J. (2011). *CBP60g* and *SARD1* play partially redundant critical roles in salicylic acid signaling. Plant J..

[53] Sun T., Busta L., Zhang Q., Ding P., Jetter R., Zhang Y. (2018). TGACG-BINDING FACTOR 1 (*TGA1*) and *TGA4* regulate salicylic acid and pipecolic acid biosynthesis by modulating the expression of SYSTEMIC ACQUIRED RESISTANCE DEFICIENT 1 (*SARD1*) and CALMODULIN-BINDING PROTEIN 60g (*CBP60g*). New Phytol..

[54] Ding Y., Sun T., Ao K., Peng Y., Zhang Y., Li X., Zhang Y. (2018). Opposite roles of salicylic acid receptors *NPR1* and *NPR3*/*NPR4* in transcriptional regulation of plant immunity. Cell.

[55] Zhang Y., Tessaro M.J., Lassner M., Li X. (2003). Knockout analysis of *Arabidopsis* transcription factors *TGA2*, *TGA5*, and *TGA6* reveals their redundant and essential roles in systemic acquired resistance. Plant Cell.

[56] Fu Z.Q., Yan S., Saleh A., Wang W., Ruble J., Oka N., Mohan R., Spoel S.H., Tada Y., Zheng N. (2012). *NPR3* and *NPR4* are receptors for the immune signal salicylic acid in plants. Nature.

[57] Petersen M., Brodersen P., Naested H., Andreasson E., Lindhart U., Johansen B., Nielsen H.B., Lacy M., Austin M.J., Parker J.E. (2000). *Arabidopsis* MAP kinase 4 negatively regulates systemic acquired resistance. Cell.

[58] Brodersen P., Petersen M., Nielsen H.B., Zhu S., Newman M.A., Shokat K.M., Rietz S., Parker J., Mundy J. (2006). *Arabidopsis* MAP kinase 4 regulates salicylic acid- and jasmonic acid/ethylene-dependent responses via *EDS1* and *PAD4*. Plant J..

[59] Wang Y., Meng Y., Zhang M., Tong X., Wang Q., Sun Y., Quan J., Govers F., Shan W. (2011). Infection of *Arabidopsis thaliana* by *Phytophthora parasitica* and identification of variation in host specificity. Mol. Plant Pathol..

[60] Jin J.-H., Zhang H.-X., Tan J.-Y., Yan M.-J., Li D.-W., Khan A., Gong Z.-H. (2016). A new ethylene-responsive factor *CaPTI1* gene of pepper (*Capsicum annuum* L.) involved in the regulation of defense response to *Phytophthora capsici*. Front. Plant Sci..

[61] Wang J.E. (2013). Expression Analysis and Functional Identification of CaRGA1 and CaPOD Genes Induced by Phytophthora Capsici in Pepper.

[62] Zhang Z., Li D.-W., Jin J.-H., Yin Y.-X., Zhang H.-X., Chai W.-G., Gong Z.-H. (2015). VIGS approach reveals the modulation of anthocyanin biosynthetic genes by *CaMYB* in chili pepper leaves. Front. Plant Sci..

[63] Ahmed S.S., Gong Z., Ji J., Yin Y., Xiao H., Khan M.A. (2012). Construction of the intermediate vector pVBG2307 by incorporating vital elements of expression vectors pBI121 and pBI221. Genet. Mol. Res..

[64] Mou S.L., Liu Z.Q., Gao F., Yang S., Su M.X., Shen L., Wu Y., He S.L. (2017). *CaHDZ27*, a homeodomain-leucine zipper I (HD-Zip I) protein, positively regulates the resistance to *Ralstonia solanacearum* infection in pepper. Mol. Plant-Microbe Interact..

[65] Zhang Y.L., Jia Q.L., Li D.W., Wang J.E., Yin Y.X., Gong Z.H. (2013). Characteristic of the Pepper *CaRGA2* Gene in Defense Responses against *Phytophthora capsici* Leonian. Int. J. Mol. Sci..

[66] Oh S.K., Jeong M.P., Young H.J., Lee S., Chung E., Kim S.Y., Seung H.Y., Choi D. (2005). A plant EPF-type zinc-finger protein, *CaPIF1*, involved in defence against pathogens. Mol. Plant Pathol..

[67] Li S., Zhao H., Li Y., Niu S., Cai B. (2012). Complete genome sequence of the naphthalene-degrading *Pseudomonas putida* strain *ND6*. J. Bacteriol..

[68] Zhao H., Chen D., Li Y., Cai B. (2005). Overexpression, purification and characterization of a new salicylate hydroxylase from naphthalene-degrading *Pseudomonas* sp. strain *ND6*. Microbiol. Res..

[69] Zhang Y.L. (2009). Identification of Physiological Race of Phytophthora Capsici and Analysis of the Efficiency of Chemical Control.

[70] Zhang H., Ali M., Feng X., Jin J., Huang L., Khan A., Lv J., Gao S., Luo D., Gong Z. (2018). A novel transcription factor *CaSBP12* gene negatively regulates the defense response against *Phytophthora capsici* in Pepper (*Capsicum annuum* L.). Int. J. Mol. Sci..

[71] Guo W.L., Chen R.G., Gong Z.H., Yin Y.X., Ahmed S.S., He Y.M. (2012). Exogenous abscisic acid increases antioxidant enzymes and related gene expression in pepper (*Capsicum annuum*) leaves subjected to chilling stress. Genet. Mol. Res..

[72] Schmittgen T.D., Livak K.J. (2008). Analyzing real-time PCR data by the comparative CT method. Nat. Protoc..

[73] Yin Y.X., Wang S.B., Zhang H.X., Xiao H.J., Jin J.H., Ji J.J., Jing H., Chen R.G., Arisha M.H., Gong Z.H. (2015). Cloning and expression analysis of *CaPIP1-1* gene in pepper (*Capsicum annuum* L.). Gene.

[74] Du Y., Mpina M.H., Birch P.R.J., Bouwmeester K., Govers F. (2015). *Phytophthora* infestans RXLR effector AVR1 interacts with exocyst component Sec5 to manipulate plant immunity. Plant Physiol..

[75] Feng X.H., Zhang H.X., Ali M., Gai W.X., Cheng G.X., Yu Q.H., Yang S.B., Li X.X., Gong Z.H. (2019). A small heat shock protein *CaHsp25.9* positively regulates heat, salt, and drought stress tolerance in pepper (*Capsicum annuum* L.). Plant Physiol. Biochem..

