# Supplementary material for: CaSBP11 Participates in the Defense Response of Pepper to Phytophthora capsici through Regulating the Expression of Defense-Related Genes
Source: Int J Mol Sci. 2020 Nov 28;21(23):9065. doi: 10.3390/ijms21239065 (PMC7729508; doi:10.3390/ijms21239065)
Supplement: Supplementary file 1 [file ijms-21-09065-s001.pdf]

SUPPLEMENTARY TABLES AND FIGURES

Supplementary Figure S1. Phenotype and silencing efficiency of CaSBP11-silenced plant. (A) Phenotypes of CaSBP11-silenced, positive control (TRV2:*CaPDS*), and negative control (TRV2:*00*) plants. Photographs were taken forty days after injection. The diameter of the pot is 15 cm. (B)The silencing efficiency of *CaSBP11* in the silenced and negative control plants. Bars with different lower-case letters indicate significant differences at $p < 0.05$.

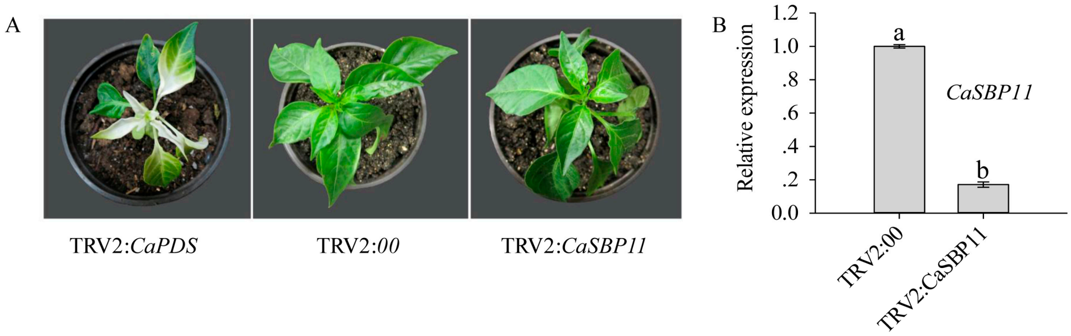

Supplementary Fiugre S2. The symptom-based scale used for the disease of CaSBP11-silenced and control plants category. level 0, no symptoms; level 1, the lower leaves of the plant turned yellow or wilted; level 2, the lower leaves of the plant have an obvious deciduous phenomenon or the whole plant wilted; level 3, blackening of stem base and all leaves have fallen off except at growth points; level 4, the whole plant is dead.

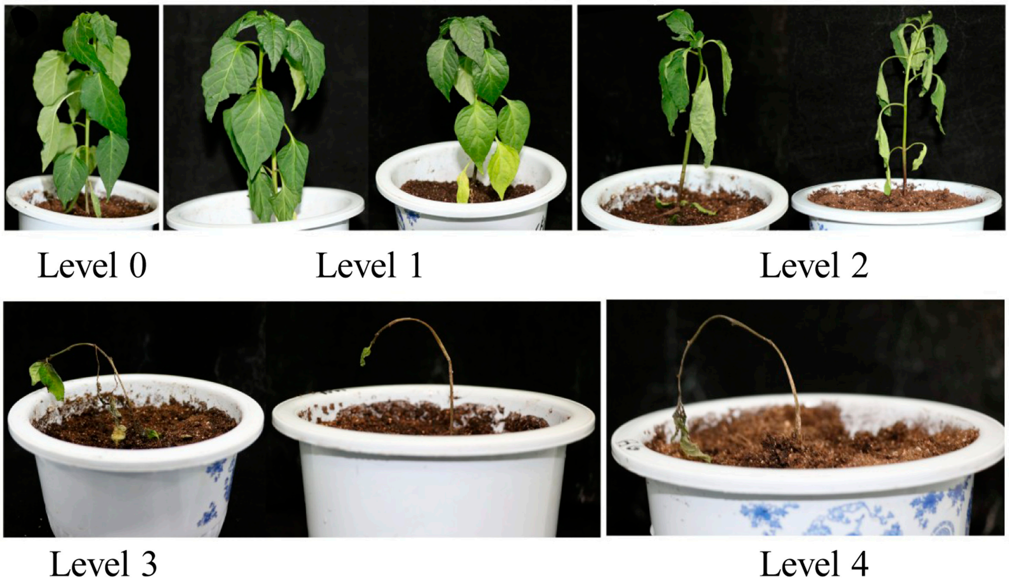

Supplementary Figure S3. Screening of *sid2-2*, *coi1-21* and *coi1-22* homozygous lines. The left primer (LP) and right primer (RP) combination and the T-DNA insertion site primer (LB1.3) and right primer (RP) combination were used to detect 12 *sid2-2* lines respectively. Only 3, 9 and 12 lines had no bands in the combination of LP and RP, while there were bands in the combination of LB1.3 and RP. Therefore, homozygous strain 3 was selected for the further study. The homozygous lines of coi1-21 and coi1-22 were screened by *Hpa*II and *BamH*I restriction endonucleases respectively. There are 3 heterozygous lines in *coi1-21* strain, and all of *coi1-22* lines are homozygous lines.

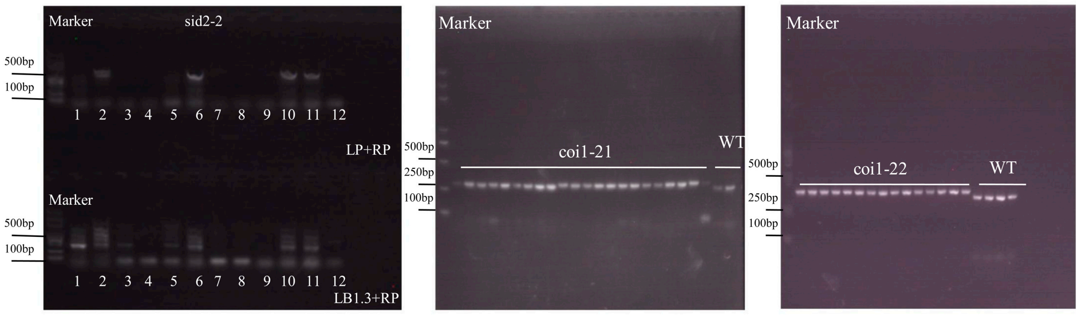

Supplementary Figure S4. Overexpression of *CaSBP11* in *Arabidopsis* and *NahG*-overexpression lines, *sid2-2*, *coi1-21,* and *coi1-22* lines. The expression levels of *CaSBP11* in these described *Arabidopsis* were detected. The expression levels of *NahG* in *NahG*-overexpression lines, and *NahG* and *CaSBP11* hybrid lines were detected. The means were analyzed using the least significant difference (LSD). * and ** represent significant differences at $p < 0.05$ and $p < 0.01$ respectively. Mean values and SDs for three replicates are shown.

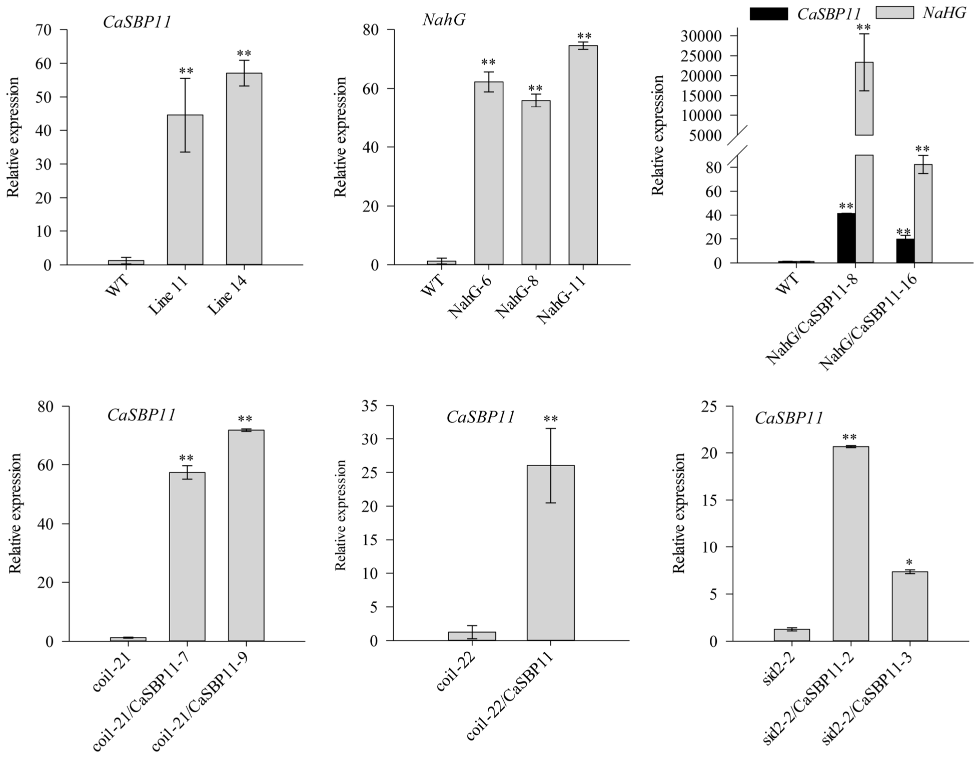

Supplementary Figure S5. Expression of the salicylic acid, jasmonate and ethylene signal pathways genes in *CaSBP11* overexpressing lines of *Arabidopsis thaliana*. The means were analyzed using the least significant difference (LSD). Bars with different lower-case letters indicate significant at $p < 0.05$. Mean values and SDs for three replicates are shown.

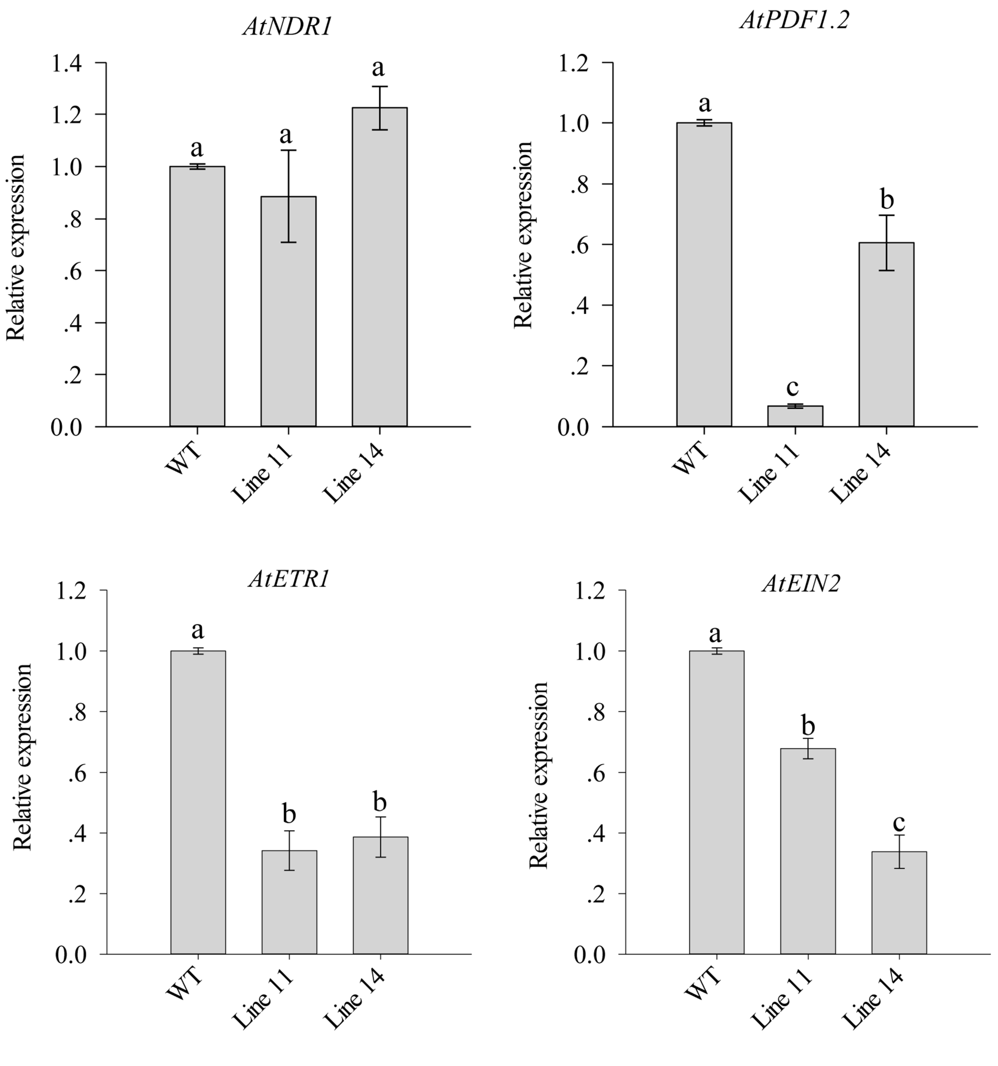

Supplementary Figure S6. Expression of the salicylic acid signaling pathway-related genes in *NahG*-overexpressing lines (NahG-6, NahG-8, and NahG-11), *NahG* and *CaSBP11* hybrid lines (NahG/CaSBP11-8 and NahG/CaSBP11-16), *sid2-2* lines and *CaSBP11* overexpression in *sid2-2* lines (sid2-2/CaSBP11-2 and sid2-2/CaSBP11-2). The means were analyzed using the least significant difference (LSD). Bars with different lower-case letters indicate significant at $p < 0.05$. Mean values and SDs for three replicates are shown.

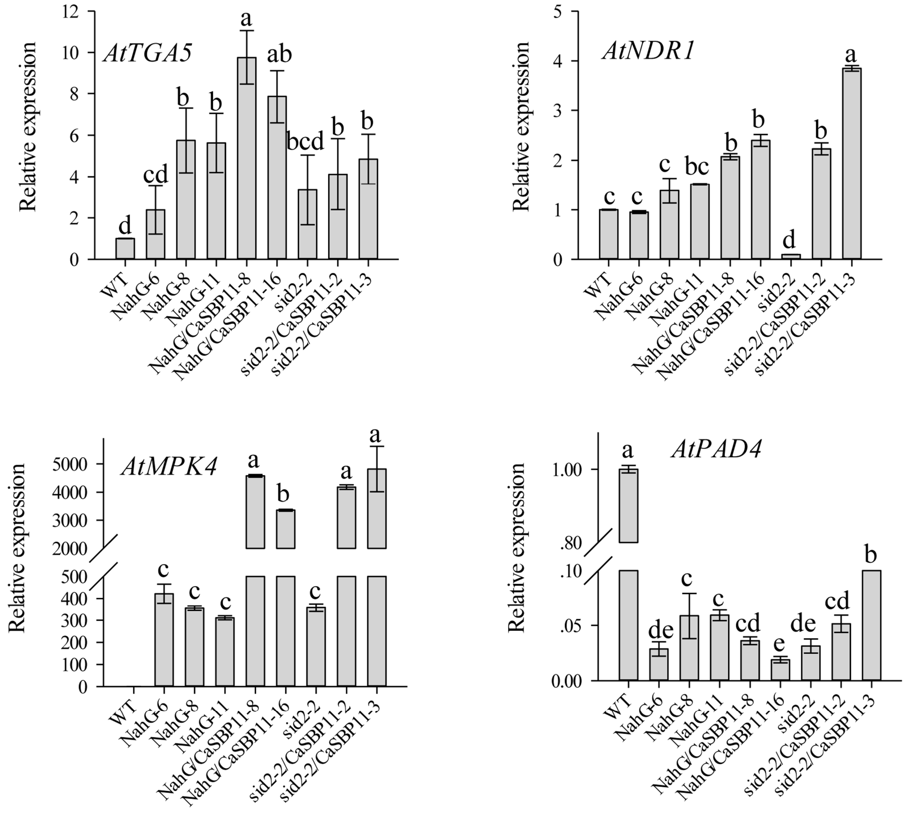

Supplementary Figure S7. Partial pattern of salicylic acid and jasmonate signalling related gene in *Arabidopsis* [38,39,51-58].Arrows indicate positive regulation, while those without arrows indicate negative regulation.

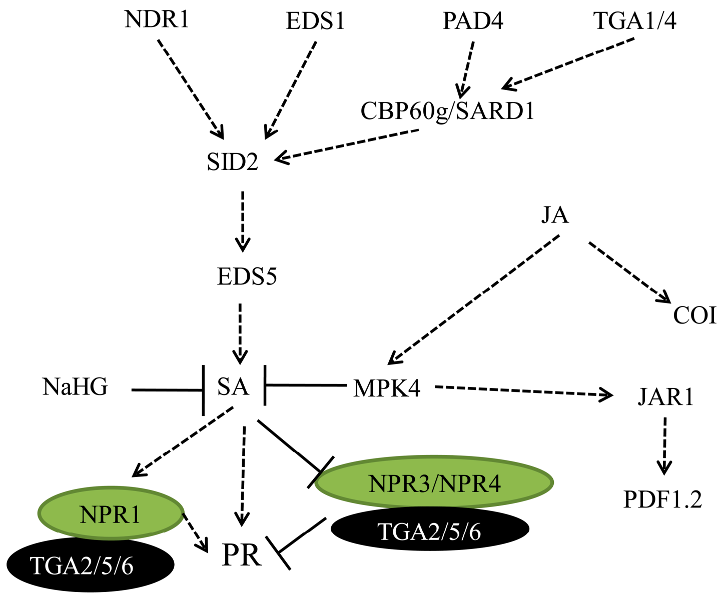

Supplementary Table S1. Primers names and their sequences used for vector construction and mutation detection in this study.

Oligo Name	Primer Abbreviation	Primer Sequence (5'-3')
CaSBP11	CaSBP11-2307-GFP-F	CGGGATCCATGGAGTCTTGGAGTTATTTCTCAGG
	CaSBP11-2307-GFP-R	TCCCCCGGGGCAGTGATTCTAAGGCCGGG
	CaSBP11-VIGS-F	CGGGATCCAGGACTGCCTGCCGTAACAAC
	CaSBP11-VIGS-R	GGGGTACCGACGAGCCCTGTGATTGAGATG
NaHG	NaHG-F	GCTCTAGAATGAAAAACAATAAACTTGGCTT
	NaHG-R	CGGGATCCTCACCCTTGACGTAGCGC
coi1-21	CS68754-F	GACAACACTTGTTGTTTTTCTTCAGACAAGGAATGTAACCG
	CS68754-R	GGTCGAGTAAGACAAGGCGGAAGTCACAGAGGTT
coi1-22	CS68755-F	CTGTAAGCAGTTGAAGCGGCTGAGGATTGAA
	CS68755-R	GTCTCAGATAGAATGCAAATCGTCTGAGTTTCTTGGAT
sid2-2	LB1.3-F	GTTCCGAAATCGGCAAAAT
	LP	AAATTTTGGGGAAATTGTTGC
	RP	ATTGAAGTGAAGCCATTGCAG

Supplementary Table S2. Primers names and their sequences used in this study for quantitative PCR.

Oligo Name	Primer Abbreviation	Primer Sequence (5'-3')
CaSBP11	RTCaSBP11-VIGS-F	CATCTCAATCACAGGGCTCG
	RTCaSBP11-VIGS-R	CATTACTATCCTGCTTCACTTGC
Nbactin-97	NbACTIN-F	TATGGAAACATTGTGCTCAGTGG
	NbACTIN-R	CCAGATTCGTCATACTCTGCC
CaActin2	CaActin2-F	TCCACCTCTTCACTCTCTGCTC
	CaActin2-R	TGACCCATCCCTACCATAACAC
CaPO1	CAPO1-F	GGCGCCAGGATTGCTGACAA
	CAPO1-R	GTGGACATAATCCTCGAAGC
CaDEF1	CADEF-F	CAAGGGAGTATGTGCTAGTGAGAC
	CADEF-R	TGCACAGCACTATCATTGCATAC

CaSAR8.2	CASAR82-F	CAGGGAGATGAATTCTGAGGC
	CASAR82-R	CATATGAACCTCTATGGATTTCTG
CaBPR1	CAPR1-F	CAGGATGCAACACTCTGGTGG
	CAPR1-R	ATCAAAGGCCGGTTGGTC
NbDEF	NbDEF-RT-F	AACTTGTGAGTCCCAGAG
	NbDEF-RT-R	GGATACCTTTCTACCACC
NbNPR1	NbNPR1-RT-F	TTACTTCACTGAAACGCCT
	NbNPR1-RT-R	CACTTCCTTTAATTCCACCT
NbPR1a	NbPR1a-RT-F	GTAATATCCCACTCTTGCCG
	NbPR1a-RT-R	ATGAAATCGCCACTTCCCTC
NbPR1b	NbPR1b-RT-F	TCAAGCTCAAAACTCTCCCC
	NbPR1b-RT-R	CCACATCTTTACTGCTCCCG
NaHG	RTnahg-F	TGCGTAGTCATGTGCTGGAAG
	RTnahg-F	GTTCGGCTTCGGCTCACTA
AtNPR1	AtNPR1-F	CGTCGCTACCGATAACAC
	AtNPR1-R	AACCGACTTCGTAATCCTT
AtNPR3	AtNPR3-F	TTCACGGGTTTGTCACCTCC
	AtNPR3-R	TCGACCAGTCTCAACTGTTTTC
AtNPR4	AtNPR4-F	TTCCCAGCAGAAGCCAATGT
	AtNPR4-R	TTCCCAGCAGAAGCCAATGT
AtPR1	AtPR1-F	ACGGGGAAAACTTAGCCTGG
	AtPR1-R	TTGGCACATCCGAGTCTCAC
AtPAD4	AtPAD4-F	TATGGTCGACGCTGCCATAC
	AtPAD4-R	CACGTGGCAGAAGTTGTGTG
AtEDS1	AtEDS1-F	GAAGAAGCAGGAGCAGTCGT
	AtEDS1-R	CCACAGAAGCTTGAAATGAGGT
AtEIN2	AtEIN2-F	CAAATGCACAACCGCAGTCA
	AtEIN2-R	CGCTTCAAAACCGAAGCCAA
AtMPK4	AtMPK4-F	AAGCTCGGGTGATCAAAGCA

	AtMPK4-R	ATTTGAGCCCACGCAACAAC
AtEDS5	AtEDS5-F	TTCGGTCCTTGGGCTGTTAC
	AtEDS5-R	CTGTGAAGCAGTTGTTGCCC
AtETR1	AtETR1-F	CCGGGGTCGAAAACTACCAA
	AtETR1-R	GGTTTGAGCAACACACCGTC
AtPDF1.2	AtPDF1.2-F	TCTCTTTGCTGCTTTCGACG
	AtPDF1.2-R	CCCTGACCATGTCCCACTTG
AtNDR1	NDR1-F	TGAAGACACAGAAGGTGGTCG
	NDR1-R	GTCTTTTCCGAGGGCAGGAA
AtSARD1	SARD1-F	TCCTCTCGCCACATCAACAC
	SARD1-R	GGCTCGCAGCATATTGTTGG
AtCBP60G	CBP60G-F	TGCCATGGATTGCGTTTTGG
	CBP60G-R	GGATCCAAACTTCCTTGAAAGTCG
AtTGA2	AtTGA2-F	AATGCGCATGCAGGTGATTC
	AtTGA2-R	TTGAACCAAGAGTCCCGCTC
AtTGA5	AtTGA5-F	AGGGCATTTGGGTATCGGTG
	AtTGA5-R	GCTTTTCCTTGCAGCCTCAC
AtTGA6	AtTGA6-F	GAGCAAGACAGCAGGGAGTT
	AtTGA6-R	ATGGGCTCTAGCTGATTCGC
AtTGA4	AtTGA4-F	TCGGATCTTAACCACGCGAC
	AtTGA4-R	TACGTTGGTTCACGTTGCCT
AtActin2	AtActin2-F	CGCTCTTTCTTTCCAAGCTCAT
	AtActin2-R	GCAAATCCAGCCTTCACCAT
